# Fasting and Caloric Restriction Activate an ADIOL‐NHR‐91‐Kynurenine Pathway Signaling Axis to Promote Healthspan

**DOI:** 10.1111/acel.70496

**Published:** 2026-04-22

**Authors:** Ana Guijarro‐Hernández, Shinja Yoo, George A. Lemieux, Sena Komatsu, Abdullah Q. Latiff, Rishika R. Patil, Kaveh Ashrafi

**Affiliations:** ^1^ Department of Physiology University of California, San Francisco San Francisco California USA

**Keywords:** androstenediol, caloric restriction, estrogen receptor beta, fasting, healthy aging, kynurenic acid, learning, movement, steroids

## Abstract

The steroid hormone 5‐androstene‐3β,17β‐diol (ADIOL) was discovered nearly a century ago in humans, yet its physiological functions have remained poorly understood. Using *C. elegans* , we identify ADIOL as essential for several pro‐healthspan effects of fasting and caloric restriction (CR). These dietary restriction regimens activate an ADIOL–NHR‐91–kynurenic acid signaling axis, partly through transcriptional programs associated with ADIOL biosynthesis. Within this axis, ADIOL acts through NHR‐91, a *C. elegans* homolog of estrogen receptor β, to reduce levels of kynurenic acid, a neuromodulatory metabolite, thereby enhancing healthspan. Critically, ADIOL does not extend lifespan, indicating its healthspan benefits are independent of longevity, and even late‐life supplementation is effective. Collectively, this work establishes ADIOL as a physiological link between metabolic cues and neural function, promoting health during aging via the kynurenine pathway. Given that in mammals ADIOL similarly is a ligand for estrogen receptor β and the kynurenine pathway influences neuroprotection mechanisms, ADIOL may represent an evolutionarily conserved signal by which dietary interventions enhance healthy aging.

## Graphical abstract

The steroid hormone ADIOL emerges as a key mediator of fasting–and caloric restriction–induced healthspan benefits in *C. elegans* . These regimens activate ADIOL production, which acts through the estrogen receptor β homolog NHR‐91 to reduce neural kynurenic acid levels and enhance healthspan independently of lifespan. ADIOL supplementation, even late in life, remains beneficial. These findings reveal a physiological role for ADIOL in linking metabolic status to neural function and suggest an evolutionarily conserved mechanism that promotes healthy aging.

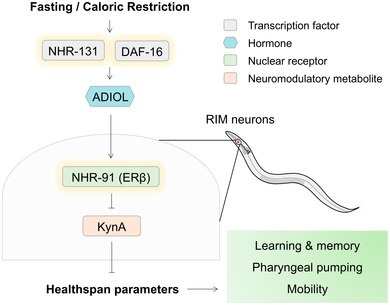

## 1 Introduction

Steroid hormones are lipophilic molecules derived from cholesterol that regulate a wide range of physiological processes. Classic examples include cortisol, a glucocorticoid that modulates stress responses, metabolism, and immune function; aldosterone, a mineralocorticoid controlling salt and water balance; and the sex steroids testosterone, an androgen, and 17β‐estradiol (E2), the principal estrogen, which govern sexual development and numerous other physiological functions (Cole et al. 2019). Among these, the steroid hormone 5‐androstene‐3β,17β‐diol (ADIOL) was discovered in humans nearly a century ago but has received extremely limited attention since.

Like other steroid hormones, ADIOL synthesis begins with cholesterol. Subsequent stepwise enzymatic actions by CYP11A1, CYP17A1, and 17β‐hydroxysteroid dehydrogenases (17β‐HSDs) convert cholesterol into ADIOL (Luu‐The 2013). The immediate precursor of ADIOL is dehydroepiandrosterone (DHEA), which is synthesized in both the mammalian gonads and adrenal glands. DHEA is also a precursor for testosterone and estrogens, including E2 (Luu‐The 2013; Figure S1A). Thus far, ADIOL has been generally regarded as a minor intermediate in the biosynthesis of sex steroids.

While initially classified as an androgen, ADIOL exhibits minimal androgenic activity (~0.21% of testosterone) (Coffey 1988). A 1997 study showed that ADIOL binds to both estrogen receptor β (ERβ) and estrogen receptor α (ERα), with ~3‐fold higher affinity for ERβ (Kuiper et al. 1997), establishing it as an estrogenic compound. ERα and ERβ are two transcription factors of the nuclear hormone superfamily that serve as molecular receptors of E2, with ERα mediating many of the classical consequences of E2 (Paterni et al. 2014; Matthews and Gustafsson 2003; Dubal et al. 2001). Unlike E2, which shows strong sex dimorphism in circulating levels during the timeframe between puberty and menopause, ADIOL is present at relatively similar levels in males and females (Manolagas et al. 1979; Li and Kannan 2022). Although ADIOL binds ERα and ERβ with only ~6% and ~17% of the affinities of E2, respectively (Kuiper et al. 1997), it is considered an endogenous ERβ ligand because its circulating levels are approximately two to three times higher than those of E2 (Lasley et al. 2011). Furthermore, astrocytes and microglia can locally convert circulating DHEA into ADIOL (Saijo et al. 2011), suggesting a mechanism for tissue‐specific regulation of ADIOL versus E2 signaling through the control of local concentrations of these two hormones. Crucially, ADIOL and E2 differ in their downstream effects upon binding ERβ. ADIOL, but not E2, suppresses microglial inflammation by recruiting CtBP corepressor complexes to AP‐1‐dependent promoters via ERβ (Saijo et al. 2011), demonstrating functionally distinct transcriptional outcomes. Thus, despite binding the same molecular receptor, ERβ, ADIOL is not simply an E2 substitute.

We recently discovered that *C. elegans* also synthesizes ADIOL (Lemieux et al. 2023). As in mammals, ADIOL biosynthesis in *C. elegans* depends on cytochrome P450 enzymes and 17β‐HSDs (Lemieux et al. 2023). These enzymes are transcriptionally regulated by NHR‐131, which is prominently expressed in the intestine, a metabolically active tissue in *C. elegans* (Lemieux et al. 2023). We found that elevating ADIOL levels enhanced the pharyngeal pumping rate, the mechanism of food intake in *C. elegans* , and improved associative learning (Lemieux et al. 2023). The specific associative learning paradigm in which ADIOL is effective requires *C. elegans* orthologs of NMDA and AMPA classes of glutamatergic receptors, as well as CaMKII kinase and CREB, a transcription factor. These molecular mechanisms play critical roles in mammalian learning or memory paradigms (Vohra et al. 2017; Vohra et al. 2018; Arey et al. 2018; Lakhina et al. 2015; Torayama et al. 2007; Kauffman et al. 2010).

Supporting the hypothesis that ADIOL acts via ERβ‐like signaling, its effects on pumping rate and learning require NHR‐91, a *C. elegans* homolog of ERβ. In *nhr‐91* mutants, ADIOL has no effects on pumping or learning behaviors, but restoring wild‐type (WT) *nhr‐91* expression specifically in RIM neurons fully rescues ADIOL responsiveness (Lemieux et al. 2023). Notably, ADIOL's effects on pharyngeal pumping and learning are not mimicked by E2 or testosterone (Lemieux et al. 2023), highlighting the functional specificity of ADIOL. Importantly, we showed that ADIOL exerts its effects on pharyngeal pumping and associative learning by reducing levels of kynurenic acid, KynA, a tryptophan‐derived neuromodulatory metabolite (Lemieux et al. 2023).

The *C. elegans* RIM neurons are interneurons that integrate external sensory cues with the animal's internal physiological state to shape behavioral outputs (White et al. 1986; Gordus et al. 2015). We previously showed that RIM neurons are among the few neurons that produce KynA (Lemieux et al. 2015; Vohra et al. 2017). Furthermore, we showed that RIM‐derived KynA regulates pharyngeal pumping and associative learning (Lemieux et al. 2015; Vohra et al. 2017). In both cases, KynA exerts its effects through inhibition of the activity of neurons that express the N‐methyl D‐aspartate class of ionotropic glutamate receptors, NMDARs: in the case of associative learning, the effects of KynA reduction depend on NMDARs in the RIM neurons, and in the case of pharyngeal pumping on NMDARs in the AVA neurons (Lemieux et al. 2015; Vohra et al. 2017; Figure S1B,C). The AVA neurons are cholinergic command interneurons that secrete FLP‐18, a neuropeptide Y‐like molecule previously shown to regulate pharyngeal pumping (Pereira et al. 2015; Rogers et al. 2003; Lemieux et al. 2023; Vohra et al. 2017). The AVA and RIM neurons are connected to each other by both chemical and electrical connections (White et al. 1986; Sordillo and Bargmann 2021; Li et al. 2023). Although KynA has been shown to act as a competitive antagonist of mammalian NMDARs, the precise mechanisms by which KynA inhibits the activity of NMDAR‐expressing neurons remain poorly understood (Stone et al. 2024). The link between KynA and learning capacity is not limited to *C. elegans* : similar to *C. elegans* , in mammals, lower KynA levels are associated with improved cognition, whereas elevated KynA impairs learning and memory (Potter et al. 2010; Moroni et al. 1988; Alexander et al. 2012; Chess et al. 2009, 2007; Chess and Bucci 2006; DeAngeli et al. 2015; Gramsbergen et al. 1992).

Although ADIOL has been recognized in humans for nearly as long as estradiol and testosterone, there is a major gap in understanding its physiological functions. Here, we provide genetic, molecular, and behavioral evidence suggesting that ADIOL biosynthesis is enhanced in response to a short‐term fast or caloric restriction (CR), two subtypes of dietary restriction. Given the well‐established links between dietary restriction, healthspan, and lifespan, we then investigated the role of ADIOL in these processes. We discovered that ADIOL is required for several of the effects of fasting and CR in the promotion of healthspan. The beneficial effects on healthspan are achieved without significant alterations to lifespan, pointing to this steroid hormone as an intervention that reduces the healthspan‐lifespan gap. Unlike most other interventions, ADIOL can improve healthspan even when its administration is initiated in aged animals. Finally, we show that ADIOL's healthspan effects depend on its ability to activate NHR‐91/ERβ signaling and the resulting reduction of neural KynA levels.

## 2 Results

### 2.1 ADIOL Links Nutrient Status to Pharyngeal Pumping and Learning Capacity

The discovery of ADIOL in *C. elegans* was facilitated by a synthetic compound, F17, initially identified through an unrelated phenotypic screen (Lemieux et al. 2023, 2011). Given the synthetic nature of F17, we sought to identify physiological conditions that depend on ADIOL activity. Because treatment of *C. elegans* with ADIOL reduces KynA levels, and KynA levels are nutritionally sensitive (Vohra et al. 2017; Lemieux et al. 2015), we hypothesized that nutritional status may modulate ADIOL signaling activity. Neural KynA levels provide a mechanism through which *C. elegans* senses nutrient availability and adjusts pharyngeal pumping accordingly (Lemieux et al. 2015): *C. elegans* rapidly reduce pumping upon food removal and increase it when reintroduced to food. Notably, if *C. elegans* animals undergo a brief 2‐h fast before refeeding, they exhibit a transient hyperactivation of pumping. Therefore, *C. elegans* increase food ingestion upon experiencing a period of fasting. This behavioral pattern is regulated by KynA levels. Fasting‐induced KynA reduction leads to NMDAR‐dependent activation of AVA neurons, triggering a neuropeptide Y‐like signaling axis (FLP‐18 neuropeptide to NPR‐5 receptor), which promotes pharyngeal pumping via serotonergic signaling (Lemieux et al. 2015). KynA‐deficient animals show elevated pumping even when well‐fed, while animals that cannot sufficiently reduce KynA levels fail to exhibit the post‐fast pumping spike (Lemieux et al. 2015).

**FIGURE 1:**
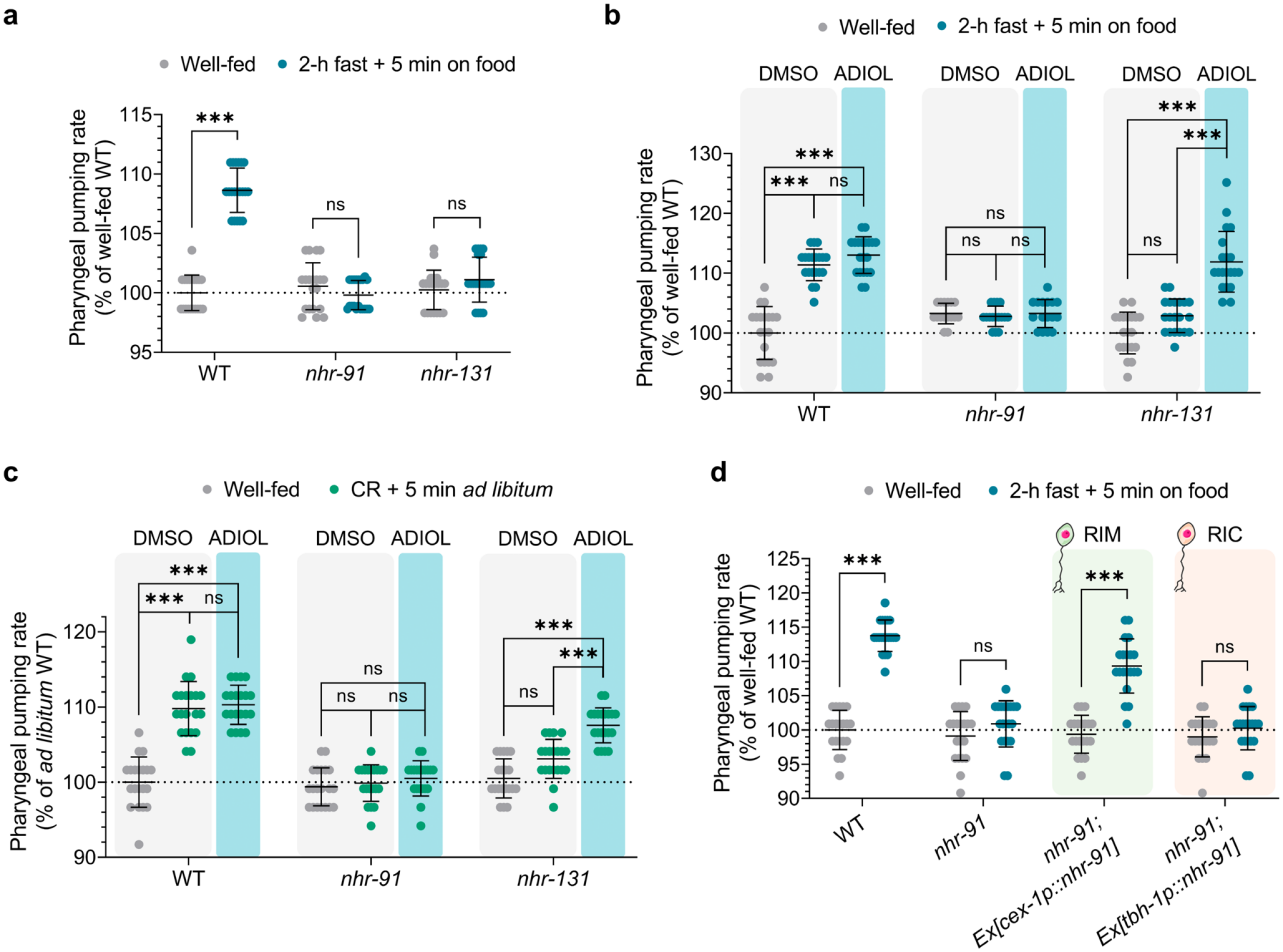
ADIOL is required for the effects of fasting and CR on pharyngeal pumping rate. (A–D) Pharyngeal pumping rate in L4‐stage animals during fed and post‐fasting or post‐CR conditions, normalized to well‐fed WT. Mean ± SD shown (*n* = 20 animals/condition). (A) WT, *nhr‐91*, and *nhr‐131* mutants. Statistics: *t*‐test (***p < 0.001, ns: non‐significant). (B, C) Same genotypes treated with DMSO or 10 nM ADIOL from the L1 stage. Statistics: one‐way ANOVA followed by Tukey's multiple comparisons test (***p < 0.001, ns: non‐significant). (D) WT, *nhr‐91*, and rescue strains (*nhr‐91; Ex[cex‐1p::nhr‐91,unc‐122::GFP]*, and *nhr‐91; Ex[tbh‐1p::nhr‐91cDNA::sl2::GFP]*) treated with DMSO or 10 nM ADIOL from the L4 stage. Statistics: *t*‐test (***p < 0.001, ns: non‐significant).

To determine if ADIOL contributes to fasting‐induced KynA reduction, we examined pharyngeal pumping after fasting in animals lacking either NHR‐91 (the ADIOL receptor, homologous to mammalian ERβ) or NHR‐131 (required for ADIOL biosynthesis in response to the synthetic compound F17). Unlike WT animals, *nhr‐91* and *nhr‐131* mutants failed to exhibit the hyperactivated pumping response after fasting (Figure 1A). To confirm that the phenotype was due to ADIOL deficiency, we administered exogenous ADIOL to *nhr‐131* and *nhr‐91* mutant animals. This fully restored the post‐fasting pumping response to WT levels in *nhr‐131* but not in *nhr‐91* mutants (Figure 1B), consistent with the roles of NHR‐131 and NHR‐91 in ADIOL biosynthesis and response, respectively. To assess whether the patterns observed upon a 2‐h fast relate to the effects of CR, we repeated the experiments in animals grown overnight on reduced bacterial food concentrations. Similar to a 2‐h fast, animals subjected to CR increased their pharyngeal pumping upon re‐exposure to plentiful food supplies in an *nhr‐91*‐ and *nhr‐131*‐dependent manner, and this behavioral deficit could be fully rescued by exogenous ADIOL in *nhr‐131* mutants, but not *nhr‐91* mutants (Figure 1C).

The *C. elegans nhr‐91* has a relatively limited expression pattern that includes the RIM and RIC interneurons (Lemieux et al. 2023), as well as some non‐neural cells (Kasuga et al. 2013). Both RIM and RIC neurons have been shown to regulate pharyngeal pumping (Greer et al. 2008), but only RIM neurons can generate KynA (Vohra et al. 2017; Lemieux et al. 2015). To determine the site of ADIOL signaling in the regulation of pharyngeal pumping, we used neuron‐specific promoters to reconstitute *nhr‐91* expression exclusively in RIM, RIC, or both neurons in *nhr‐91* mutants. ADIOL increased pumping when *nhr‐91* was expressed in both RIM and RIC neurons or exclusively in RIM neurons, but not when expressed in RIC neurons without concomitant expression in RIM (Figure S2A), pinpointing RIM neurons as the site of ADIOL action. Consistent with this, the presence of *nhr‐91* in RIM but not in RIC neurons was sufficient to restore the post‐fasting pumping response to WT levels (Figure 1D), highlighting the role of *nhr‐91* in the RIM neurons in regulating a physiologically relevant function. We confirmed that the promoters used in these reconstitution studies correctly target the indicated neurons by imaging the expression of fluorescent reporters driven by each promoter. In all cases, we observed the expected expression patterns (Figure S2B), with no deviations from those previously reported (Alkema et al. 2005; Wang et al. 2024; Taylor et al. 2025).

As we previously demonstrated through direct biochemical measurements that ADIOL reduces KynA levels, which underlies its enhancement of pharyngeal pumping, these findings support a model in which fasting and CR activate NHR‐131 to promote ADIOL biosynthesis, which then binds to NHR‐91 in RIM neurons to reduce KynA levels and thereby enhance pharyngeal pumping.

To assess whether ADIOL's effects are sexually dimorphic, we measured pharyngeal pumping in hermaphrodites and males treated with ADIOL or F17, which elevates ADIOL levels (Lemieux et al. 2023). Both compounds enhanced pumping in both sexes (Figure S2C), indicating that ADIOL acts independently of biological sex.

**FIGURE 2:**
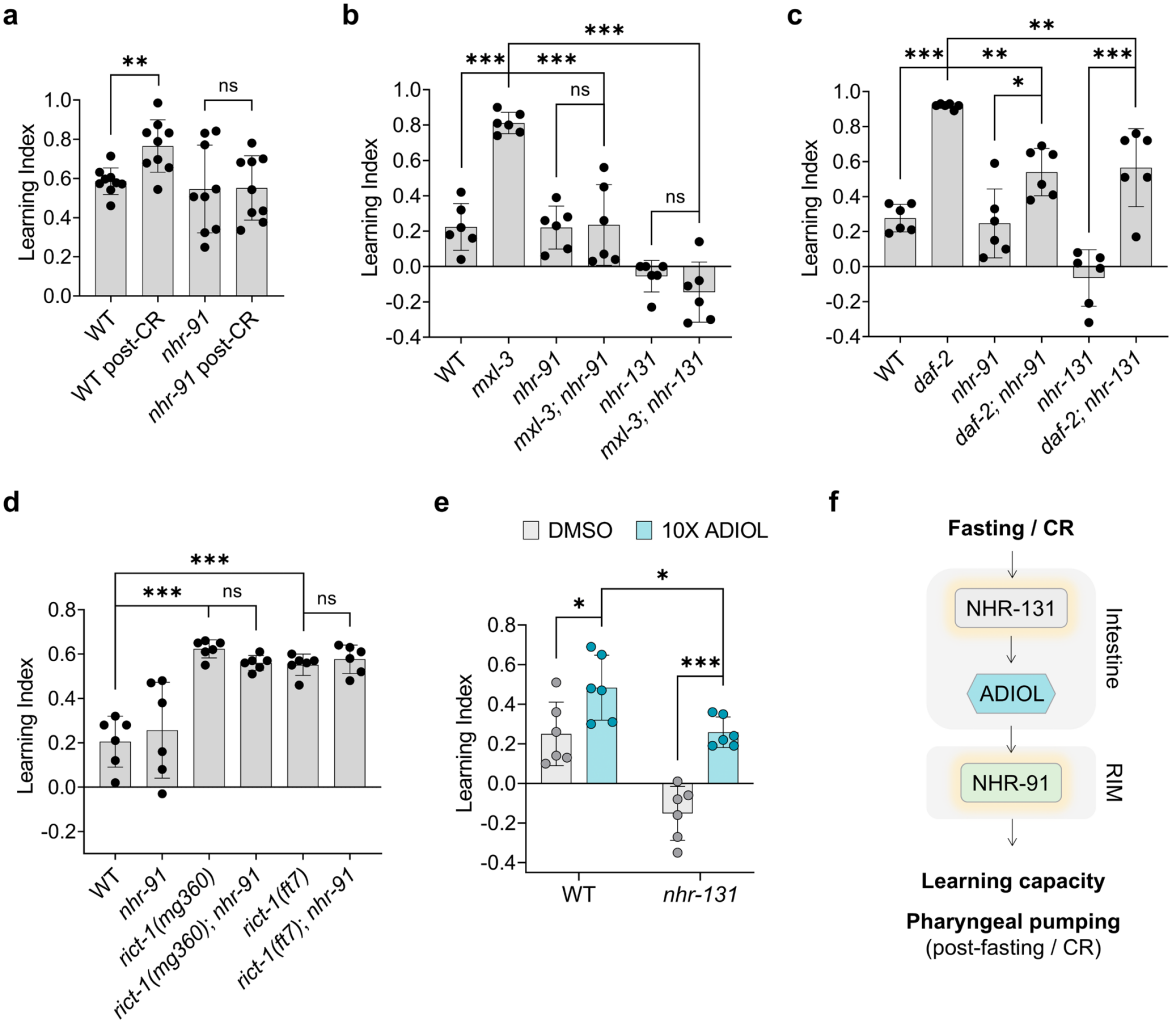
ADIOL contributes to the beneficial effects of CR on learning capacity. (A–E) Effects of various genotypes and nutrient conditions on learning indices of Day 1 adult animals. Mean ± SD shown. (A) Well‐fed animals and animals subjected to overnight CR (*n* = 9; 26–117 animals/replicate). Statistics: *t*‐test (**p < 0.01, ns: non‐significant). (B–D) Genetic models of CR, *mxl‐3* (B) and *daf‐2* (C), and loss‐of‐function mutant alleles of the non‐CR genetic model *rict‐1* (D), analyzed in the presence or absence of *nhr‐91* or *nhr‐131* (*n* = 6; 15–332 animals/replicate). Statistics: one‐way ANOVA followed by Tukey's multiple comparisons test (***p < 0.001, **p < 0.01, *p < 0.05, ns: non‐significant). (E) WT and *nhr‐131* mutant animals treated from the L1 stage with either DMSO or 100 nM ADIOL (10X) (*n* = 6; 112–344 animals/replicate). Statistics: *t*‐test (***p < 0.001, *p < 0.05, ns: non‐significant). (F) Model illustrating the regulatory connection between nutrient conditions, learning capacity, and pharyngeal pumping via ADIOL. Fasting and CR activate NHR‐131 to promote ADIOL production, which subsequently activates NHR‐91 in the RIM neurons to enhance both learning capacity and pharyngeal pumping.

Prior work showed that treating *C. elegans* with ADIOL promotes learning and memory in *C. elegans* via reductions in KynA (Lemieux et al. 2023). While both NMDAR‐dependent and independent learning paradigms exist, the benefits of reduced KynA are specific to *C. elegans* learning paradigms that are NMDAR‐dependent (Vohra et al. 2017). To determine whether ADIOL signaling plays a role in learning regulation in a physiologically relevant condition, we examined the effects of CR. Consistent with previous reports (Vohra et al. 2017), using an NMDAR‐dependent associative learning paradigm, we first showed that CR enhances learning capacity (Figure 2A). We then showed that this enhancement is abolished in *nhr‐91* mutants, even though under well‐fed conditions, WT and *nhr‐91* mutants had relatively similar learning capacities (Figure 2A).

We next examined two well‐established genetic mutants, *mxl‐3* and *daf‐2*, because they mimic some aspects of CR. MXL‐3 is a transcription factor whose activity represses lysosomal lipolysis and autophagy (O'Rourke and Ruvkun 2013). In turn, starvation represses *mxl‐3* expression, which leads to increased autophagy (O'Rourke and Ruvkun 2013). These mutants exhibit reduced KynA levels, as determined by biochemical assays, even when well fed (Vohra et al. 2017). They also show elevated pharyngeal pumping, dependent on the same signaling components required for the increased pumping observed in KynA‐deficient *nkat‐1* mutants (Figure S2D), as well as enhanced learning (Figure 2B) (Vohra et al. 2017). We found that the learning enhancement in *mxl‐3* mutants was entirely dependent on the presence of *nhr‐91* and *nhr‐131* (Figure 2B). Consistent with prior data, *nhr‐91* mutants showed near‐WT learning, while *nhr‐131* mutants exhibited significant deficits (Lemieux et al. 2023).

The *C. elegans* *daf‐2* encodes a receptor kinase with sequence and functional homologies to mammalian insulin receptor and insulin‐like growth factor 1 receptor (Kimura et al. 1997). Loss‐of‐function mutations in *daf‐2* mimic aspects of CR, and the *daf‐2* mutants, similar to *mxl‐3* mutants, were previously shown to have reduced KynA levels and enhanced learning (Vohra et al. 2017). Losses of *nhr‐91* and *nhr‐131* partially suppressed learning enhancement of *daf‐2* mutants (Figure 2C).

We next sought to assess whether ADIOL signaling through NHR‐91 is generally involved in the regulation of learning behavior or has a more specific role in linking metabolic status through a 2‐h fast or CR to learning capacity. We had previously found that *rict‐1* mutants are KynA deficient and exhibit enhanced learning capacity (Vohra et al. 2017). RICTOR, encoded by *rict‐1*, is the defining component of mechanistic Target Of Rapamycin Complex‐2, mTORC‐2. While inhibition of mTORC‐1 is frequently used to mimic aspects of CR, that is not the case for mTORC‐2 inhibition. We validated the previous finding that *rict‐1* mutants have elevated learning capacity relative to WT animals; however, this enhanced learning was not dependent on *nhr‐91* (Figure 2D). Thus, reductions of KynA levels can be achieved independently of ADIOL signaling.

Given that NHR‐131 regulates multiple steroidogenic enzymes, including CYP17A1/CYP‐13A4, a key regulatory node likely to affect biosynthesis of multiple steroids, the more severe learning deficits of *nhr‐131* mutants compared to *nhr‐91* mutants likely reflect a broader role for NHR‐131 in steroidogenesis. To determine whether the learning deficits of *nhr‐131* mutants are at least partly due to ADIOL deficiency, we exposed these animals to high concentrations of ADIOL and observed a partial rescue in Day 1 adults (Figure 2E). We have previously shown that *nhr‐91* mutants are unresponsive to ADIOL in this learning assay (Lemieux et al. 2023).

Together, these results reveal requirements for ADIOL signaling under physiologically relevant conditions. They also indicate that while multiple mechanisms can contribute to reductions in KynA and its subsequent effects on learning capacity, ADIOL signaling plays a prominent role under conditions of CR (Figure 2F).

### 2.2 Fasting and CR Increase Transcription Levels of Genes Implicated in ADIOL Biosynthesis

**FIGURE 3:**
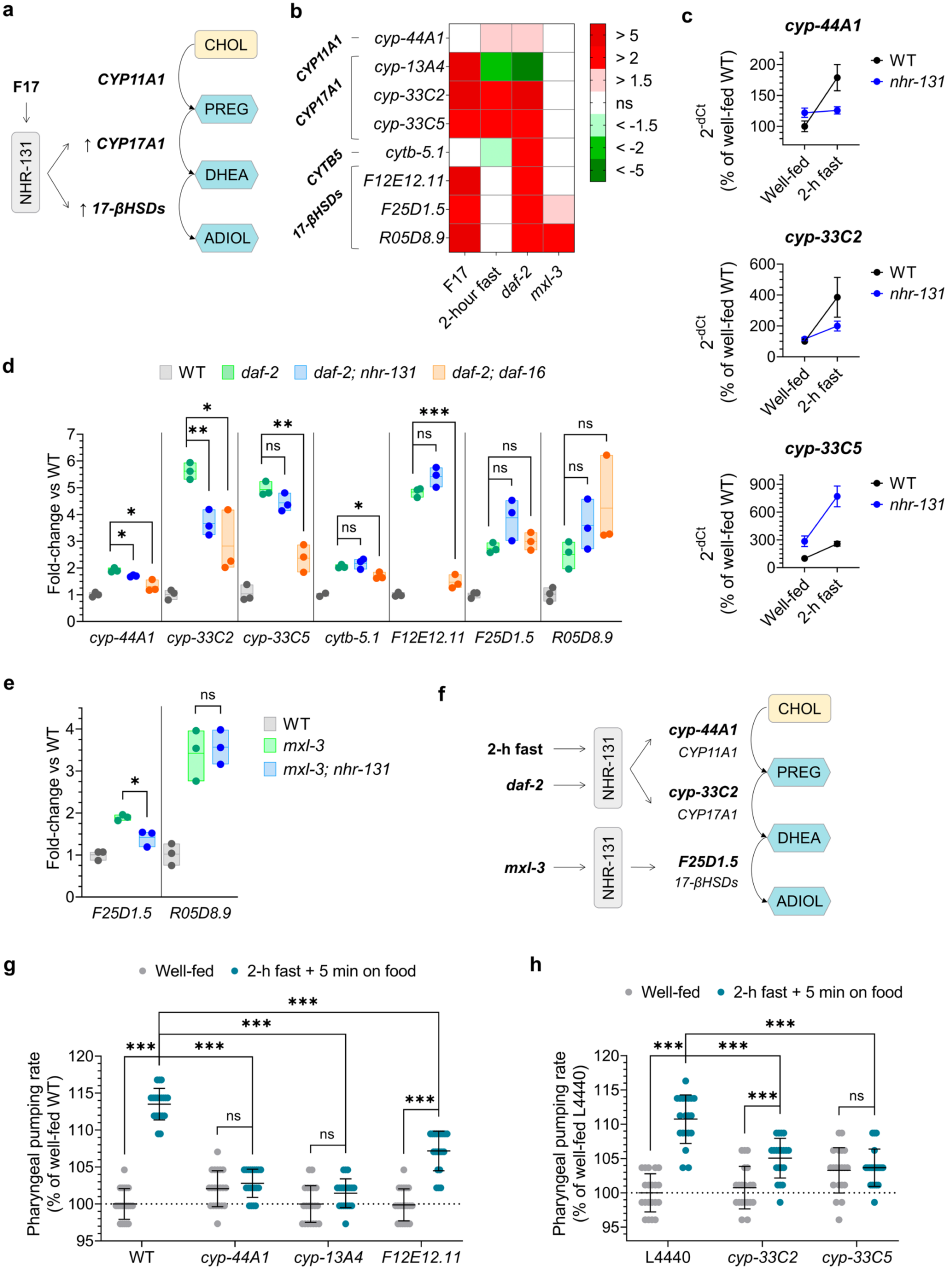
Fasting and genetic models of CR upregulate subsets of ADIOL biosynthesis genes. (A) Schematic indicating that F17‐induced activation of NHR‐131 promotes ADIOL biosynthesis from cholesterol by upregulating *C. elegans* homologs of human CYP17A1 and 17‐βHSDs, which catalyze the conversion of pregnenolone (PREG) to dehydroepiandrosterone (DHEA), and DHEA to ADIOL, respectively (Lemieux et al. 2023). (B) RT‐qPCR analysis of *CYP11A1*, *CYP17A1*, and *17β‐HSD* homologs in L4 animals treated with 2.5 μM F17 or under conditions shown (*n* = 3; ~300 animals/replicate). Mean ± SD fold‐change values shown relative to DMSO‐treated controls (for F17‐treated animals) or to well‐fed WT animals (for 2‐h fasting and the mutants shown). Statistics: one‐way ANOVA followed by Dunnett's test on Δ*C*_t_ values. Non‐statistically significant differences (ns) are shown in white. (C) RT‐qPCR of *cyp‐44A1*, *cyp‐33C2*, and *cyp‐33C5* in WT and *nhr‐131* L4 animals under fed and fasting conditions (*n* = 3; ~300 animals/replicate). Mean ± SD values shown relative to well‐fed WT. Statistics: two‐way ANOVA on Δ*C*_t_ values (positive interaction for *cyp‐44A1* (*p* = 0.0004) and *cyp‐33C2* (*p* = 0.02); no interaction for *cyp‐33C5* (*p* = 0.89)). (D, E) RT‐qPCR analysis of a subset of ADIOL biosynthesis genes in *daf‐2* (D) and *mxl‐3* (E) mutants deficient in *nhr‐131* or *daf‐16* (*n* = 3; ~300 animals per condition). Mean, minimum, and maximum values shown relative to WT. Statistics: *t*‐test on Δ*C*_t_ values (***p < 0.001, **p < 0.01, *p < 0.05, ns: not significant). (F) Model showing the dependence on *nhr‐131* for the upregulation of ADIOL biosynthetic genes in the context of fasting and CR. (G, H) Pharyngeal pumping rate in L4‐stage animals under fed and post‐fasting conditions (*n* = 20 animals/condition). Mean ± SD values shown relative to well‐fed WT. Statistics: *t*‐test (***p < 0.001, ns: non‐significant). (G) WT, *cyp‐44A1*, *cyp‐13A4*, and *F12E12.11* animals were analyzed. (H) WT animals exposed to RNAi clones L4440, *cyp‐33C2*, or *cyp‐33C5* from the L1 stage.

In mammals, ADIOL is synthesized from cholesterol via a well‐characterized enzymatic cascade: CYP11A1 converts cholesterol to pregnenolone (PREG); CYP17A1 transforms PREG into 17α‐hydroxypregnenolone, which is then converted to dehydroepiandrosterone (DHEA) by the combined actions of CYP17A1 and its partner CYTB5, a cytochrome b5 reductase. Next, 17β‐HSDs convert DHEA to ADIOL (Figure 3A). Prior work showed that the synthetic compound F17 increases ADIOL levels in *C. elegans* . This effect of F17 requires the intestinally expressed NHR‐131 transcription factor (Lemieux et al. 2023). RNA‐seq followed by functional and biochemical analyses led to the identification of a set of F17‐upregulated genes dependent on *nhr‐131* that encode the *C. elegans* homologs of *CYP17A1*, *CYTB5*, and *17β‐HSDs* (Lemieux et al. 2023). As is often the case in metabolic pathways, we found multiple genes as homologs of *CYP17A1* and *17β‐HSDs*. Furthermore, based on sequence homology, we identified the *C. elegans* *cyp‐44A1* as a likely homolog of *CYP11A1*, encoding the enzyme that catalyzes the conversion of cholesterol to pregnenolone. To determine whether the transcriptional activities of these genes respond not only to F17, a synthetic compound, but also to physiologically relevant conditions such as fasting or CR models, we analyzed their expression levels using RT‐qPCR.

Compared to the levels seen in well‐fed WT animals, both fasting and loss of *daf‐2* induced the expression of *cyp‐44A1* (*CYP11A1*), and *cyp‐33C2* and *cyp‐33C5* (*CYP17A1* homologs) (Figure 3B). In the case of *daf‐2*, *cytb‐5.1* was also upregulated (Figure 3B). By contrast, none of these genes were upregulated in *mxl‐3* mutants (Figure 3B). Further analysis revealed that the fasting‐induced changes in *cyp‐44A1* and *cyp‐33C2* expression were dependent on *nhr‐131* (Figure 3C), and in the case of *daf‐2* mutants, also dependent on *daf‐16*, the main downstream effector of *daf‐2* (Figure 3D). All three *17β‐HSD* homologs showed elevated expression in *daf‐2* mutants (Figure 3B), two were also upregulated in *mxl‐3* mutants, and none upon a 2‐h fast (Figure 3B). Among these, only the upregulation of *F25D1.5* in *mxl‐3* mutants was NHR‐131–dependent (Figure 3E).

Thus, while distinct expression patterns were found, at least a subset of ADIOL biosynthetic pathway genes was upregulated, in some cases via *nhr‐131*, under conditions where ADIOL levels are predicted to be elevated (Figure 3F).

We reasoned that if these biosynthetic genes contribute to ADIOL production, their losses should mimic the effects of impaired ADIOL signaling. To test this, we examined post‐fast pharyngeal pumping. *cyp‐44A1* and *cyp‐13A4* mutants failed to elevate pharyngeal pumping following refeeding, while *F12E12.11* mutants showed only a partial response (Figure 3G). Similarly, animals exposed to RNAi knockdown of *cyp‐33C2* and *cyp‐33C5* (*CYP17A1* homologs) failed to fully activate pumping upon post‐fast refeeding (Figure 3H). These partial requirements likely reflect enzymatic redundancy, with multiple enzymes capable of catalyzing each step.

Together, the finding that a subset of genes involved in ADIOL biosynthesis exhibit increased expression upon fasting or in genetic models of CR, and in some cases in an *nhr‐131*‐dependent manner, is consistent with functional data indicating that ADIOL signaling is activated under these conditions. These results also reveal the complexity of metabolic pathways, both in terms of redundancy and in the precise ways they are regulated by each nutrient‐sensing mechanism, as illustrated by *daf‐2* and *mxl‐3* mutants, as well as by 2‐h fasting. It is highly likely that additional transcriptional and post‐transcriptional regulatory mechanisms play pivotal roles in ADIOL production.

### 2.3 ADIOL Signaling Does Not Appreciably Affect Lifespan

**FIGURE 4:**
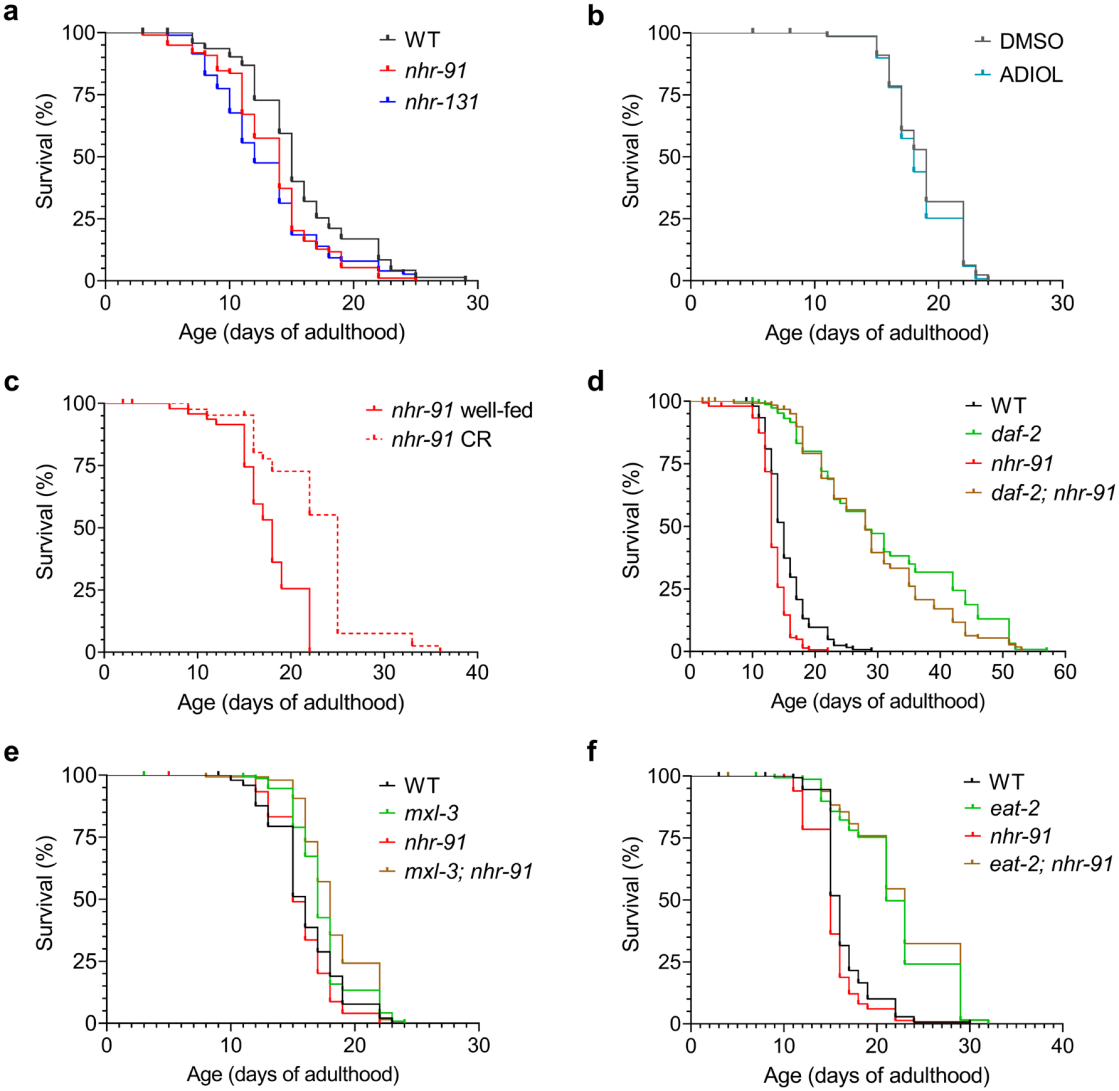
ADIOL has minimal effects on longevity. (A) Lifespan of WT, *nhr‐91*, and *nhr‐131* animals at 20°C. Median survival: 15 days (WT, *n* = 100), 14 days (*nhr‐91*, *n* = 99; −6.7% vs. WT), and 12 days (*nhr‐131*, *n* = 96; −20% vs. WT). Log‐rank test with Bonferroni correction showed significant differences between WT and *nhr‐91* (*p* = 0.002) and between WT and *nhr‐131* (*p* = 0.001). (B) Lifespan of WT animals treated with DMSO or 10 nM ADIOL from the L4 stage. Median survival: 19 days (DMSO, *n* = 136) and 18 days (ADIOL, *n* = 150; −5.3% vs. DMSO). No significant differences were detected (log‐rank test, *p* > 0.01). (C) Lifespan of well‐fed or caloric‐restricted *nhr‐91* animals. Median survival: 18 days (AL, *n* = 50) and 25 days (CR, *n* = 50; +38.9% vs. WT). CR significantly extended lifespan (log‐rank test, *p* < 0.0001). (D–F) Lifespan curves of the CR genetic models *daf‐2* (D), *mxl‐3* (E), and *eat‐2* (F) deficient in *nhr‐91*. Kaplan–Meier survival curves were compared using the log‐rank test with Bonferroni correction. (D) Median survival was 15 days (WT, *n* = 154), 28 days (*daf‐2*, *n* = 150; +86.7% vs. WT), 13 days (*nhr‐91*, *n* = 151; −13.3% vs. WT), and 28 days (*daf‐2; nhr‐91*, *n* = 150; 0% vs. *daf‐2*). No significant differences between *daf‐2* and *daf‐2; nhr‐91* mutants (*p* > 0.01). (E) Median survival was 16 days (WT, *n* = 150), 17 days (*mxl‐3*, *n* = 150; +6.3% vs. WT), 15 days (*nhr‐91*, *n* = 150; −6.3% vs. WT), and 18 days (*mxl‐3; nhr‐91*, *n* = 150; +5.9% vs. *mxl‐3*). No significant differences between *mxl‐3* and *mxl‐3; nhr‐91* mutants (*p* > 0.01). (F) Median survival was 16 days (WT, *n* = 150), 21 days (*eat‐2*, *n* = 150; +31.3% vs. WT), 15 days (*nhr‐91*, *n* = 150; −6.3% vs. WT), and 23 days (*eat‐2; nhr‐91*, *n* = 150; +9.5% vs. *eat‐2*). No significant differences between *eat‐2* and *eat‐2; nhr‐91* mutants (*p* > 0.01).

Exposure of WT animals to CR, as well as mutations in *daf‐2* or *mxl‐3*, extend lifespan in *C. elegans* (Klass 1977; O'Rourke and Ruvkun 2013; Kimura et al. 1997). To evaluate the role of ADIOL in longevity, we examined *nhr‐91* and *nhr‐131* mutants. Animals lacking the putative ADIOL receptor (*nhr‐91* mutants) exhibited a modest reduction in median lifespan (approximately 1 day shorter than WT), whereas *nhr‐131* mutants, which are predicted to be deficient in the biosynthesis of multiple steroid hormones, including ADIOL, displayed a more substantial decrease (Figure 4A). ADIOL supplementation did not extend the lifespan of WT animals (Figure 4B).

Moreover, CR achieved by reducing bacterial food availability extended lifespan in *nhr‐91* mutants, and loss of *nhr‐91* did not diminish the longevity of long‐lived *daf‐2*, *mxl‐3*, or *eat‐2* mutants (Figure 4C–F). Animals with loss‐of‐function mutations in *eat‐2* exhibit reduced food intake due to defects in pharyngeal pumping and have thus been used as a model of CR, primarily because of their extended lifespan (Raizen et al. 1995; Lakowski and Hekimi 1998).

Thus, although the effects of CR on pharyngeal pumping and learning capacity are dependent on ADIOL signaling, this signaling pathway is dispensable for CR‐mediated lifespan extension. This is consistent with prior findings showing that reductions in KynA levels do not affect lifespan (Vohra et al. 2017).

### 2.4 ADIOL Promotes Healthspan During Aging Through KynA Reduction

**FIGURE 5:**
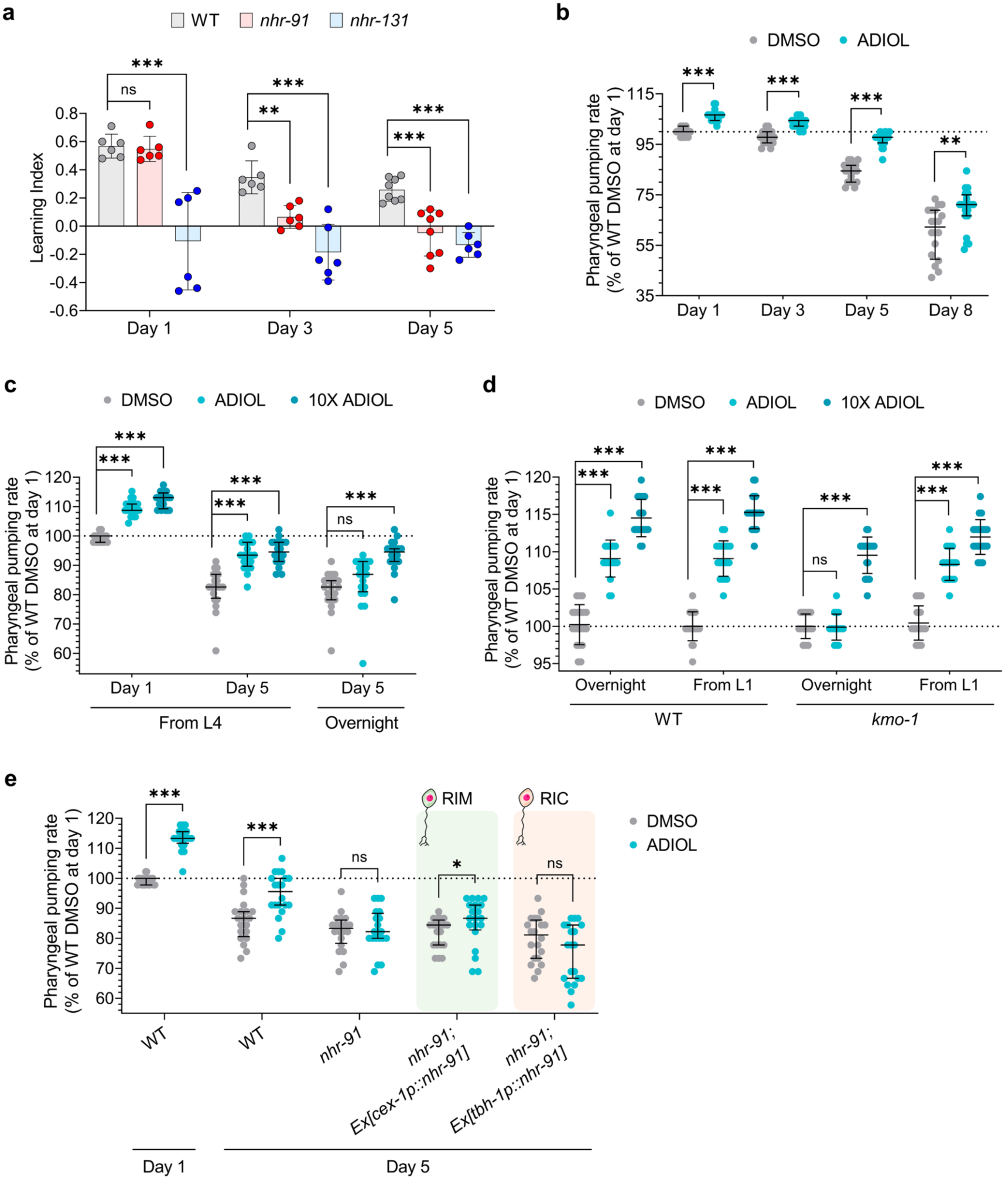
ADIOL promotes pharyngeal pumping and learning, two metrics of healthspan, during aging. (A) Learning index of WT, *nhr‐91*, and *nhr‐131* animals on Day 1, 3, and 5 of adulthood (*n* = 6–8; 25–232 animals/replicate) after exposure to 50 μM FUDR from Day 1. Mean ± SD shown. Statistics: one‐way ANOVA followed by Dunnett's test (***p < 0.001, **p < 0.01, ns: non‐significant). (B, C) Pharyngeal pumping rate during aging (*n* = 20 animals/condition). Animals were exposed to 50 μM FUDR from Day 1. Median ± IQR values shown relative to WT DMSO‐treated Day 1 adults. (B) WT animals treated with DMSO or 10 nM ADIOL from the L4 stage, assayed on Days 1, 3, 5, and 8 of adulthood. Statistics: *t*‐test (for Day 1 and Day 3 adults) or Mann–Whitney *U*‐test (for Day 5 and Day 8 adults) (***p < 0.001, **p < 0.01). (C) WT animals treated with DMSO, 10 nM ADIOL, or 100 nM ADIOL (10×) on Days 1 and 5 of adulthood. For Day 5 adults, treatment began either at the L4 stage or on Day 4 (overnight). Statistics: one‐way ANOVA followed by Dunnett's test (for Day 1 adults) or Mood's median test followed by Mann–Whitney *U*‐test with Bonferroni correction (for Day 5 adults) (***p < 0.001, ns: non‐significant). (D) Pharyngeal pumping rate in Day 1 WT and *kmo‐1* animals treated with DMSO, 10 nM ADIOL, or 100 nM ADIOL (10X), with treatments initiated at either the L4 or L1 stage (*n* = 20 animals/condition). Data are shown as percentage relative to overnight DMSO‐treated WT controls (mean ± SD). Statistics: one‐way ANOVA followed by Dunnett's test (***p < 0.001, ns: non‐significant). (E) Pharyngeal pumping rate on Day 5 adults of WT, *nhr‐91*, *nhr‐91; Ex[cex‐1p::nhr‐91,unc‐122::GFP]*, and *nhr‐91; Ex[tbh‐1p::nhr‐91cDNA::sl2::GFP]* treated with DMSO or 10 nM ADIOL from the L4 stage (*n* = 20 animals/condition). Animals were exposed to 50 μM FUDR from Day 1. Median ± IQR values shown relative to WT DMSO‐treated Day 1 adults. Statistics: *t*‐test or Mann–Whitney *U*‐test depending on distribution (***p < 0.001, *p < 0.05, ns: non‐significant).

Healthspan refers to the period of lifespan during which an organism maintains robust physiological function (Khan et al. 2024). Several metrics have been measured as indicators of healthspan in *C. elegans* (Hwang et al. 2025; Kauffman et al. 2010; Keith et al. 2014). Among these are learning capacity and pharyngeal pumping, two parameters affected by ADIOL in young animals. To systematically assess the effects of ADIOL on these healthspan parameters, we first asked if ADIOL is required to maintain normal learning capacity throughout aging. While WT animals exhibited age‐associated declines in learning, *nhr‐91* mutants, which are unable to respond to ADIOL, showed a markedly earlier and more pronounced decline (Figure 5A). Similarly, *nhr‐131* mutants, which lack ADIOL biosynthesis, displayed severe impairments from early adulthood (Figure 5A). The more pronounced phenotype of *nhr‐131* mutants is, once again, consistent with the notion that this transcription factor likely exerts control over levels of steroid hormones beyond ADIOL.

We also found that, relative to WT animals, *nhr‐91* and *nhr‐131* mutants exhibited more rapid age‐associated declines in pharyngeal pumping rate (Figure S3A). In turn, we found that treatment of WT animals with ADIOL from the L4 stage led to enhanced pharyngeal pumping at every age tested (Figure 5B).

To determine whether ADIOL administration remains effective after the onset of functional decline, we treated Day 4 adult animals with either the standard dose or a tenfold higher dose of ADIOL and measured pumping on Day 5. While the standard dose had no effect, the higher dose restored pumping to levels comparable to those seen with lifelong treatment (Figure 5C). Day 5 animals were previously demonstrated to have significantly elevated levels of KynA (Vohra et al. 2018), providing a potential rationale as to why elevated doses of ADIOL are needed to elicit an increase in pharyngeal pumping.

To further test this model, we treated WT animals and *kmo‐1* mutants, which exhibit elevated levels of KynA even on Day 1 (van der Goot et al. 2012; Lemieux et al. 2015), with low and high doses of ADIOL for varying durations. All subsequent measurements were performed on Day 1 adults. WT animals showed increased pumping rates in response to both low and high ADIOL doses, regardless of whether treatment was administered overnight or initiated at the L1 stage (extended exposure). In contrast, KynA‐replete *kmo‐1* mutants were resistant to overnight treatment with low‐dose ADIOL (Figure 5D). Importantly, this resistance was overcome either by overnight exposure to high‐dose ADIOL or by prolonged treatment beginning at earlier developmental stages, both of which significantly increased pumping rates in *kmo‐1* mutants (Figure 5D).

To determine whether ADIOL's effects on pumping remain dependent on RIM neurons in aged animals, we examined *nhr‐91‐*deficient animals in which *nhr‐91* was specifically expressed in RIM or RIC neurons. Only RIM‐specific reconstitution restored the ability of ADIOL to enhance pharyngeal pumping in Day 5 adults, confirming the continued importance of this neuronal locus (Figure 5E).

Since biochemical measurements of KynA extracted from whole animals indicate ADIOL treatment reduces KynA levels (Lemieux et al. 2023), the above learning and pharyngeal pumping results suggest that the beneficial effects of ADIOL are due to KynA reductions. Consistent with this, we previously reported that KynA‐deficient *nkat‐1* mutants maintain elevated pharyngeal pumping and learning capacity during aging, while *kmo‐1* mutants, shown to have elevated KynA levels, have severe deficits (Vohra et al. 2018).

**FIGURE 6:**
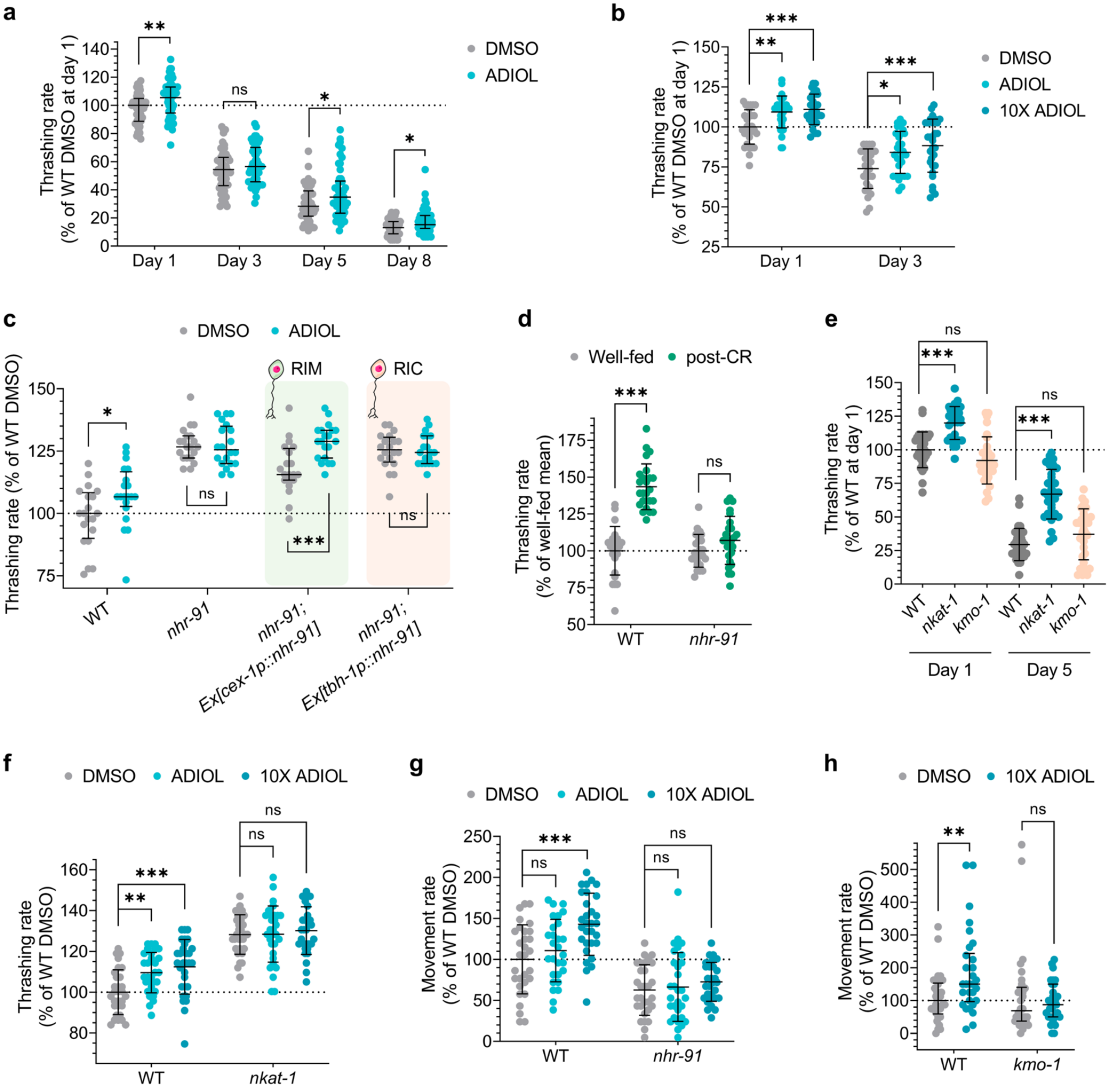
ADIOL and KynA reduction promote mobility during aging. (A, B) Thrashing rate during aging (*n* = 20–50 animals/condition). Unless otherwise indicated, animals were exposed to 50 μM FUDR starting on Day 1 and treated with DMSO or ADIOL from the L4 stage. (A) Thrashing rates on Days 1, 3, 5, and 8 of adulthood of WT animals treated with DMSO or 10 nM ADIOL. Median ± IQR values shown relative to WT DMSO‐treated Day 1 adults. Statistics: *t*‐test (Days 1, 3) or Mann–Whitney *U*‐test (Days 5, 8) (**p < 0.01, *p < 0.05, ns: non‐significant). (B) WT animals treated with DMSO, 10 nM ADIOL, or 100 nM ADIOL (10×) on Days 1 and 3. No FUDR was used in this assay. Mean ± SD values shown relative to WT DMSO‐treated Day 1 adults. Statistics: one‐way ANOVA followed by Dunnett's test (***p < 0.001, **p < 0.01, *p < 0.05). (C) Thrashing rate of Day 1 adult WT, *nhr‐91*, *nhr‐91; Ex[cex‐1p::nhr‐91,unc‐122::GFP]*, and *nhr‐91; Ex[tbh‐1p::nhr‐91cDNA::sl2::GFP]* animals treated with DMSO or 10 nM ADIOL. Data are shown as percentage relative to WT DMSO‐treated animals (median ± IQR). Statistics: Mann–Whitney *U*‐test (***p < 0.001, *p < 0.05, ns: non‐significant). (D) Thrashing rate in L4‐stage WT and *nhr‐91* mutant animals under post‐CR conditions, normalized to well‐fed controls (*n* = 25 animals/condition). Mean ± SD shown. Statistics: *t*‐test (***p < 0.001; ns, not significant). (E) Thrashing rate of WT, *nkat‐1* and *kmo‐1* animals on Day 1 and Day 5 of adulthood (*n* = 30 animals/condition). Data are shown as percentage relative to Day 1 WT adults (mean ± SD). Statistics: one‐way ANOVA followed by Dunnett's test (***p < 0.001, ns: non‐significant). (F) Thrashing rate of WT and *nkat‐1* animals on Day 1 of adulthood after DMSO, 10 nM ADIOL or 100 nM ADIOL (10X) starting at L4 stage (*n* = 20 animals/condition). Data are shown as percentage relative to WT DMSO‐treated animals (mean ± SD). Statistics: one‐way ANOVA followed by Dunnett's test (***p < 0.001, **p < 0.01, ns: non‐significant). (G) Spontaneous movement of WT and *nhr‐91* animals on Day 1 of adulthood after treatment with DMSO, 10 nM ADIOL or 100 nM ADIOL (10×) starting at the L4 stage (*n* = 20 animals/condition). Data are shown as percentage relative to WT DMSO‐treated animals (mean ± SD). Statistics: one‐way ANOVA followed by Dunnett's test (***p < 0.001, ns: non‐significant). (H) Movement rate of WT and *kmo‐1* animals on Day 1 of adulthood after DMSO or 100 nM ADIOL (10X) starting at L4 stage (*n* = 30 animals/condition). Data are shown as percentage relative to WT DMSO‐treated animals (median ± IQR). Statistics: Mann–Whitney *U*‐test (**p < 0.01, ns: non‐significant).

To examine other indicators of healthspan, we next assessed mobility by measuring thrashing, the swimming movement of worms in liquid. As expected, thrashing declined with age (Figure 6A). The age‐dependent declines in thrashing rate and increases in its variability were similar to previously reported measurements of thrashing (Bansal et al. 2015; Stuhr and Curran 2020). ADIOL treatment from the L4 stage increased thrashing in Day 1 adults, but its effects at later ages were modest and potentially obscured by the high variability of thrashing rate in the absence of treatment (Figure 6A). We considered the possibility that the muted effects may reflect the presence of the drug 5‐Fluoro‐2′‐deoxyuridine (FUDR) in the experiment. FUDR is commonly used in *C*. *elegans* aging studies as it facilitates the experimental procedure by preventing progeny development. However, FUDR is known to have additional detrimental effects (Wang et al. 2019). Therefore, we repeated the experiment without FUDR and also tested a higher dose of ADIOL. Unlike the experiments containing FUDR, ADIOL robustly enhanced thrashing in aged adults (Figure 6B). As with pharyngeal pumping, we found that ADIOL enhanced thrashing only when NHR‐91 was reconstituted in RIM neurons (Figure 6C). The RIM neurons have been shown to regulate movement (Li et al. 2023). Finally, we found that animals that have experienced CR exhibit an elevated thrashing rate when re‐exposed to plentiful food supplies (Figure 6D). This enhancement was dependent on *nhr‐91* (Figure 6D).

To further evaluate the relationships of ADIOL and KynA in thrashing, we next examined *nkat‐1* mutants (low KynA) and *kmo‐1* mutants (high KynA) (Lemieux et al. 2023; Lemieux et al. 2015). *nkat‐1* mutants exhibited elevated thrashing on both Day 1 and 5, and *kmo‐1* mutants did not further diminish the already reduced thrashing of Day 5 animals (Figure 6E). Consistent with a KynA‐dependent mechanism, ADIOL, even at a high dose, failed to further enhance thrashing in *nkat‐1* mutants (Figure 6F).

Another commonly used metric of healthspan is spontaneous animal movement on solid media in the presence of plentiful food supplies. We found that, relative to WT animals, *nhr‐91* mutants exhibited reduced movement (Figure 6G) and that ADIOL treatment increased locomotion in an NHR‐91–dependent manner (Figure 6G). Once again, consistent with the notion that ADIOL exerts its effects ultimately by reducing KynA levels, KynA‐replete *kmo‐1* mutants were resistant to the movement‐enhancing effects of ADIOL (Figure 6H).

Finally, we evaluated whether ADIOL influences other aging‐related phenotypes. ADIOL‐treated animals showed a trend toward modestly increased resistance to osmotic stress (NaCl) (Figure S3B). A similar trend, albeit not reaching statistical significance, was seen in *nkat‐1* mutants (Figure S3C). Moreover, chemotaxis to benzaldehyde was unaffected by ADIOL at any age (Figure S3D). These data indicate that ADIOL enhances multiple, but not all, indicators of healthspan.

Together, these results suggest that loss of ADIOL signaling via NHR‐91 accelerates decline in multiple healthspan indicators while exogenous ADIOL administration mitigates these declines during aging. Moreover, the previously demonstrated KynA‐reducing effects of ADIOL, together with our analyses of *nkat‐1* and *kmo‐1* mutants, support the notion that the healthspan‐enhancing effects of ADIOL are likely due to reductions in KynA levels.

### 2.5 Fasting, CR, and Loss of *Nhr‐91* Alter Kynurenine Pathway Metabolite Levels

To more comprehensively define how nutrient status influences the kynurenine pathway (KP), we examined the effects of acute fasting (2 h in the complete absence of food) and CR (18 h on a diluted bacterial food source) on KP metabolites in WT and *nhr‐91* mutant animals.

We used high‐performance liquid chromatography (HPLC) to quantify tryptophan (Trp), the initiating substrate of the KP; kynurenine (Kyn), a central branch point intermediate; and two Kyn‐derived metabolites, KynA and anthranilic acid (Ant), from whole‐animal extracts (Figure S1C). Levels of 3‐hydroxykynurenine (3‐HKyn) were below the limit of detection using this method.

**FIGURE 7:**
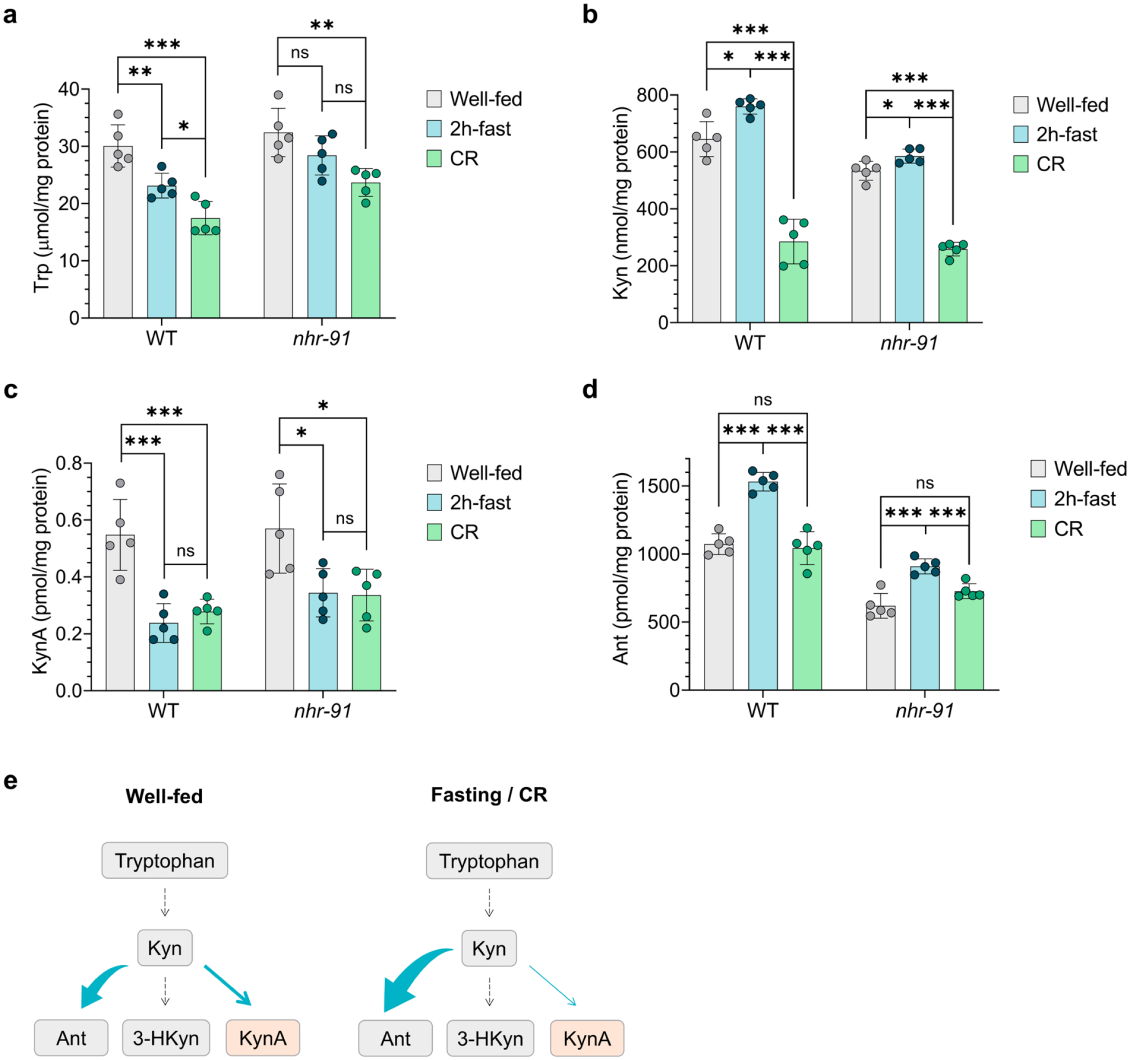
Fasting, CR, and loss of *nhr‐91* alter kynurenine pathway metabolite levels. (A–E) HPLC quantification of kynurenine pathway metabolites in WT and *nhr‐91* animals following a 2‐h fast or overnight CR (*n* = 5; ~25,000 animals/replicate). Mean ± SD shown. Statistics: one‐way ANOVA followed by Tukey's multiple comparisons test. (A) Tryptophan (TRP). (B) Kynurenine (Kyn). (C) Kynurenic acid (KynA). (D) Anthranilic acid (Ant). (F) Proposed model illustrating the effects of fasting and CR in directing Kyn toward the Ant branch of the pathway.

Across all conditions tested, and consistent with prior reports, steady‐state levels of KP metabolites differed dramatically in abundance. In well‐fed WT animals, Trp levels (~30 μmol/mg protein) were approximately 50‐fold higher than Kyn (~600 nmol/mg protein), ~30,000‐fold higher than Ant (~1000 pmol/mg protein), and ~60,000,000‐fold higher than KynA (~0.5 pmol/mg protein) (Figure 7A–D; Table S1). These measurements show the striking differences in abundance between distinct KP intermediate pools.

Under well‐fed conditions, WT and *nhr‐91* mutants exhibited similar levels of Trp (*p* = 0.38) and KynA (*p* = 0.81). In contrast, Kyn and Ant levels were significantly reduced in *nhr‐91* mutants relative to WT (Figure 7A–D; *p* = 0.007 and < 0.0001, respectively), suggesting that loss of *nhr‐91* alters basal KP flux.

In WT animals, fasting caused a significant decrease in Trp levels accompanied by increases in both Kyn and Ant (Figure 7A,B,D). Consistent with the behavioral findings, fasting also significantly reduced KynA levels (Figure 7C). Because the KP is the primary pathway for *de novo* NAD^+^ biosynthesis in animals (Yang and Sauve 2016), these results suggest that fasting mobilizes Trp into the KP. Within the 2‐h fasting window, this appears to result in accumulation of Kyn that is preferentially shunted toward Ant, the arm of the pathway that feeds into NAD^+^ synthesis or terminal oxidative metabolism, and away from KynA production (Figure 7E; Figure S1C).

In *nhr‐91* mutants, fasting similarly elevated Kyn and Ant levels, although the reduction in Trp did not reach statistical significance (Figure 7A,B,D). Importantly, the decrease in KynA was also blunted: whereas KynA levels declined by ~57% in fasted WT animals, they declined by ~40% in *nhr‐91* mutants (Figure 7C). The incomplete suppression of KynA in the absence of *nhr‐91* is consistent with the limited cellular overlap between *nhr‐91* and *nkat‐1* expressions, which prominently intersect only in the RIM neurons.

Like fasting, CR reduced Trp and KynA levels in WT animals, and these effects were again attenuated in *nhr‐91* mutants (Figure 7A,C). However, unlike fasting, CR led to a significant reduction in Kyn levels, while Ant levels remained unchanged (Figure 7B,D). One interpretation consistent with these findings is that prolonged nutrient limitation (18 h under CR vs. 2 h of fasting) results in sustained mobilization of Trp into the Kyn to Ant to NAD^+^ branch of the pathway (Figure 7E). Over time, this continued flux may drive more extensive depletion of Trp and prevent Kyn accumulation by continuously channeling it toward NAD^+^ biosynthesis. Under CR conditions, *nhr‐91* mutants qualitatively mirrored WT responses to CR, but with attenuated magnitude, further supporting a modulatory role for *nhr‐91* in regulating KP flux.

Finally, we examined KP metabolites in *daf‐2* mutants. Consistent with the established view that *daf‐2* animals under nutrient‐replete conditions mimic aspects of CR, we observed significant reductions in all four measured KP metabolites (Table S1).

Together, these findings indicate that fasting, CR, and genetic mimetics of CR converge on a common metabolic outcome: reduction of KynA levels. These data support a model in which nutrient limitation remodels KP flux in a manner that promotes healthspan‐associated phenotypes caused by KynA reductions. More broadly, the results demonstrate that KP metabolite levels are dynamically regulated not only by nutritional state but also by the duration of nutrient limitation, and they establish *nhr‐91* as a functional regulator of KP metabolism consistent with genetic and behavioral evidence on *nhr‐91* mutants. Finally, these results indicate that reductions in KynA observed under conditions of nutrient limitation are unlikely to simply reflect a lack of availability of its precursors. This is because while fasting and CR result in changes in Trp and Kyn levels, they remain at vast excess compared to KynA levels.

## 3 Discussion

Steroid hormones such as the mineralocorticoid aldosterone, the glucocorticoid cortisol, and the sex hormones estradiol and testosterone exert profound effects across diverse aspects of organismal biology. Despite longstanding knowledge of the presence of ADIOL in humans, little is known about the physiological processes in which this steroid participates. We previously found that in *C. elegans* , increasing ADIOL levels reduces KynA levels, with consequences for pharyngeal pumping and learning capacity (Lemieux et al. 2023). However, because those experiments relied on F17, a synthetic compound that promotes ADIOL generation, they did not establish a definitive link between endogenous ADIOL and physiological regulation.

In the present study, we uncover a previously unrecognized physiological role for the ADIOL signaling pathway as a nutritionally responsive regulator of behavior, including pharyngeal pumping, learning capacity, and mobility, key parameters of healthspan in *C. elegans* . We show that increasing ADIOL levels enhances these measures in both young and aging animals, whereas loss of molecular regulators of ADIOL biosynthesis or signaling prevents the beneficial effects of fasting and CR. In several contexts, disruption of ADIOL signaling accelerates age‐associated declines in these metrics. Importantly, ADIOL selectively improves healthspan without extending lifespan, indicating that its beneficial effects are not secondary to effects on lifespan.

Consistent with nutritional regulation of ADIOL biosynthesis, genes predicted to encode enzymes involved in its synthesis, including homologs of *CYP11A1*, *CYP17A1*, and *17β‐HSDs*, are upregulated following fasting or in genetic models that mimic aspects of CR. In several cases, we provide functional evidence linking these transcriptional changes to nutrient‐dependent modulation of pharyngeal pumping, a behavior also regulated by ADIOL and its receptor. Collectively, both genetic and behavioral data support the conclusion that ADIOL signaling is enhanced under nutrient‐deprived conditions. A limitation of the present study, however, is the absence of direct biochemical measurements of ADIOL, which remain technically challenging given the small size of *C. elegans* and the difficulty of measuring low‐abundance steroids in complex lysates.

Although the connection between fasting/CR and enhanced ADIOL signaling is novel, related findings in vertebrates suggest evolutionary conservation. Plasma DHEA, the precursor to ADIOL, increases during fasting in male zebra finches and *Anolis sagrei* (Fokidis et al. 2013; Himmelstein et al. 2021). In rodents, fasting induces hepatic CYP17A1 expression and elevates DHEA levels via PGC‐1α activation (Grasfeder et al. 2009; Milona et al. 2019). Prolonged CR in mice also enhances ERβ expression (Słuczanowska‐Głąbowska et al. 2015). These findings support the broader concept that steroidogenic pathways linked to ADIOL are nutritionally responsive across species.

Behavioral analyses of genetic mutants presented here, together with prior characterization of *nkat‐1* and *kmo‐1* mutants, indicate that ADIOL promotes healthspan by reducing KynA levels. Direct biochemical measurements confirm that fasting and CR, whether induced by food dilution or genetic manipulation, reduce KynA levels and identify *nhr‐91* as a functional regulator of the kynurenine pathway (KP).

Interpretation of KP metabolite measurements from whole‐animal extracts requires several considerations. First, the KP is dynamically regulated; therefore, both nutritional state and duration of exposure must be carefully considered. In our study, comparisons between well‐fed animals and those subjected to a 2‐h fast or 18 h of CR revealed time‐dependent changes consistent with activation of the anthranilic acid branch under nutrient limitation. However, definitive evaluation of pathway dynamics will require flux analyses. Second, KP intermediates differ widely in steady‐state abundance, in some cases by orders of magnitude. Thus, condition‐dependent changes likely reflect regulatory redistribution of flux between pathway branches rather than simple substrate limitation. Indeed, under nutrient deprivation, KynA levels declined despite the continued presence of relatively abundant Trp and Kyn levels. Third, KP metabolite signaling, from KynA in particular, is spatially restricted (Lemieux et al. 2015; Vohra et al. 2017), thus biochemical measurements derived from whole‐animal extracts are opaque to this critical context.

Pertaining to localized effects of KynA and its regulation by ADIOL, reconstitution experiments indicate that ADIOL's effects on pharyngeal pumping and learning primarily result from localized changes in KynA produced by the RIM interneurons. Although *nkat‐1*, which encodes an enzyme responsible for approximately half of whole‐organism KynA production (Lemieux et al. 2015; Vohra et al. 2017), and *nhr‐91* are expressed in a limited number of cells, their prominent overlap only occurs in the RIM neurons (Lemieux et al. 2023). Previous work showed that ADIOL treatment reduces KynA levels by ~30% (Lemieux et al. 2023), whereas nutrient deprivation reduces KynA by ~50%–60% (Vohra et al. 2017). These findings suggest that ADIOL contributes to, but does not fully account for, fasting‐induced KynA depletion. This is consistent with our observation that loss of *nhr‐91* blunts but does not abolish fasting and CR effects on KynA levels. Importantly, even modest changes in KynA measured from whole animals appear sufficient to influence complex behavioral outcomes, which likely reflects its spatially restricted signaling role.

The precise molecular mechanisms linking ADIOL–NHR‐91 activity in RIM neurons to changes in KynA remain unknown. Another key question is how the ADIOL–NHR‐91/ERβ–KynA signaling axis promotes healthspan. KynA's known signaling properties offer a plausible framework. We previously demonstrated that the benefits of KynA reduction on pharyngeal pumping and learning depend on enhanced neuronal activity in an NMDAR‐dependent manner (Vohra et al. 2017). However, NMDAR‐independent mechanisms may also contribute, as KynA acts in mammals as an agonist of the aryl hydrocarbon receptor (AhR) and GPR35, a G‐protein coupled receptor (DiNatale et al. 2010; Wang et al. 2006; Stone 1993).

KP metabolites such as KynA are increasingly being recognized as active signaling molecules involved in numerous pathological processes (Cervenka et al. 2017; Schwarcz et al. 2012; Schwarcz and Stone 2017; Kolodziej et al. 2011; Stone and Darlington 2013; Joisten et al. 2021). In mammals, dysregulated KP signaling has been implicated in neurodegenerative disorders. Crucially, there is compelling evidence that these altered levels are causative for cognitive defects that characterize neurodegeneration, and that correcting these altered levels provides a novel therapeutic strategy (Cervenka et al. 2017; Johnson and Macauley 2024). Additionally, KP has been linked to healthspan regulation in mammals (Kaiser et al. 2020). For instance, elevated kynurenine (Kyn), the precursor to KynA, is associated with sarcopenia (Lustgarten and Fielding 2017), hip fractures (Kim et al. 2019; Apalset et al. 2014), and cardiovascular disease (Zuo et al. 2016) in older adults, and with muscle atrophy (Kaiser et al. 2019) and bone loss (Refaey et al. 2017) in animal models. For these reasons, there is great interest in identifying interventions that affect the balance of KP metabolites. Our findings indicate ADIOL as an endogenous *C. elegans* mechanism for modulating KP metabolite levels. As ADIOL and its receptor, ERβ, are also present in mammals, our findings suggest that a similar regulatory effect may exist in mammals.

Collectively, our results establish ADIOL signaling as a link between nutritional status and behavioral health metrics, revealing a physiological function for this previously overlooked steroid hormone. By enhancing healthspan without extending lifespan, ADIOL may help narrow the healthspan–lifespan gap, a central goal in aging research. Given the emerging importance of the kynurenine pathway in mammalian age‐associated pathologies and the conserved requirement for ERβ in mediating ADIOL's effects, these findings raise the possibility that ADIOL‐based interventions could represent a general strategy for promoting healthy aging.

## 4 Materials and Methods

### 4.1 Strains and Maintenance

Strains used in this study are listed in Table S2. Plasmid constructs were generated by standard PCR amplification from *C. elegans* genomic DNA or cDNA and assembled using NEBuilder HiFi DNA Assembly (New England Biolabs). Transgenic extrachromosomal arrays included *Ex[cex‐1p::nhr‐91, unc‐122::GFP]*, in which a 0.9 kb region upstream of the *cex‐1* ATG drove the *nhr‐91* genomic sequence with *unc‐122::GFP* as a co‐injection marker; *Ex[tbh‐1p::nhr‐91 cDNA::SL2::GFP]*, containing 3455 bp upstream of the *tbh‐1* ATG driving full‐length *nhr‐91* cDNA followed by a bicistronic SL2::GFP reporter; and *Ex[tdc‐1p::nhr‐91::SL2::GFP]*, containing 2.1 kb upstream of the *tdc‐1* ATG driving *nhr‐91* genomic sequence followed by SL2::GFP. The 3′ untranslated region (UTR) of *unc‐54* was used for all constructs to ensure proper transcript processing. Transgenic animals carrying non‐integrated extrachromosomal arrays were generated by microinjecting expression plasmids into the gonad arm at final concentrations ranging from 5 to 50 ng/μL. Double mutants were obtained through standard genetic crosses between males and hermaphrodites.

Unless otherwise specified, *C. elegans* were maintained at 20 °C on 6‐cm NGM agar plates seeded with *Escherichia coli* OP50, following established protocols (Brenner 1974).

### 4.2 RNAi

RNAi clones targeting *cyp‐33C2* and *cyp‐33C5* (Ahringer library) (Kamath et al. 2003) were used for gene knockdown experiments. The empty vector L4440 was used as a negative control, and the *nkat‐1* RNAi clone was included as a positive control. *E. coli* HT115 strains containing the respective RNAi plasmids were grown overnight at 37 °C in 5 mL of LB supplemented with 100 μg/mL carbenicillin. Cultures were then diluted 1:40 into 5 mL of fresh LB with 100 μg/mL carbenicillin and grown at 37 °C with shaking until reaching an OD_600_ of 0.4–0.6. Double‐stranded RNA production was induced by adding 1 mM IPTG and incubating for 4 h at 37 °C. To spike the induction, cultures were supplemented with an additional 100 μg/mL carbenicillin and 1 mM IPTG. A total of 250 μL of induced culture was seeded onto 6‐cm NGM plates containing 100 μg/mL carbenicillin and 1 mM IPTG. Plates were incubated overnight at room temperature to allow bacterial lawn formation. Synchronized L1 animals were then transferred onto the RNAi plates and maintained at 20 °C until the L4 stage for further experiments.

### 4.3 Pharmacological Treatments

The pharmacological treatments used in this study were F17 (custom synthesis, TimTec) and 5‐androstene‐3β,17β‐diol (ADIOL) (Steraloids). Both compounds were dissolved in DMSO, which served as the vehicle. F17 was applied at a final concentration of 2.5 μM, and ADIOL was administered at a final concentration of 10 nM, with a subset of experiments including a tenfold higher dose (10X; 100 nM) to assess dose‐dependent effects. Concentrations were calculated based on the total NGM volume per plate and were mixed with *E. coli* OP50 prior to seeding. Unless otherwise specified, treatments were initiated at the L4 larval stage.

### 4.4 2‐h Fasting

Fasting was conducted by collecting animals in 1 mL of S‐basal +0.05% PEG‐8000 and washing them three times with the same solution. After washing, worms were rotated for 2 h in the same buffer. For pharyngeal pumping assessment, animals were transferred to plates seeded with *E. coli* OP50 and allowed to acclimate for at least 5 min before counting. The fasting procedure in liquid culture for metabolite determination is described in Section 4.14 of the Materials and Methods.

### 4.5 CR

CR was implemented by bacterial dilution on agar plates, with dilutions determined in pilot experiments based on animal number and developmental stage. CR conditions in liquid culture for metabolite determination are described in Section 4.14.

For pharyngeal pumping and thrashing assays, ~400 L1 animals were placed on 6‐cm NGM plates seeded with *E. coli* OP50. After 24 h at 20°C, animals were washed with 1 mL S‐basal +0.05% PEG‐8000, and ~100 animals were transferred to assay plates containing 200 μL OP50 added 1 h prior (10^9^ CFU/mL for well‐fed or 10^7^ CFU/mL for CR; plates were dried for 2 h beforehand). Animals were assayed the following day at the L4 stage. For post‐CR pharyngeal pumping and thrashing, animals were transferred to plates seeded with 200 μL OP50 (10^9^ CFU/mL) and allowed to acclimate for at least 5 min before counting.

For learning assays, ~250 L4 animals were transferred to plates containing 200 μL OP50 (10^9^ CFU/mL for well‐fed or 10^8^ CFU/mL for CR), prepared on the same day, and assayed the following day as Day 1 adults. Prior to exposure to butanone, animals were transferred to fresh plates seeded with 200 μL OP50 (10^9^ CFU/mL).

For lifespan assays, 25 day 1 adult animals were transferred to plates containing OP50 (10^10^ CFU/mL for well‐fed or 10^9^ CFU/mL for CR) supplemented with carbenicillin (100 μg/mL) and FUDR (50 μg/mL; from Day 1 to 8 of adulthood) and were moved to fresh plates every other day.

### 4.6 Pharyngeal Pumping

Pharyngeal pumping was assessed by counting the number of contractions of the posterior pharyngeal bulb over a 10‐s period, as previously described (Lemieux et al. 2015). The assay was performed on 20 L4 or Day 1 adult animals per condition. Animals were age‐synchronized using standard hypochlorite treatment of gravid hermaphrodites to isolate eggs, which were allowed to hatch overnight in S‐basal supplemented with 0.05% PEG‐8000. Approximately 150 synchronized L1 larvae per condition were transferred onto 6‐cm NGM plates and incubated at 20 °C until they reached the developmental stage required for each assay. For aging studies, Day 1 adults were placed on plates containing 50 μM FUDR (RPI) and moved to fresh FUDR plates every other day until evaluation. For details on the preparation of animals for this assay under CR conditions, please see Section 4.5 of the Materials and Methods.

### 4.7 Short‐Term Associative Learning

Short‐term associative learning was performed as previously described (Lemieux et al. 2023) with minor modifications. Animals were age‐synchronized via bleaching and allowed to hatch overnight in S‐basal supplemented with 0.05% PEG‐8000. Approximately 250 L1 larvae per condition and replicate were transferred onto 6‐cm NGM plates and incubated at 20 °C until they reached the appropriate developmental stage for the assay. For aging studies, Day 1 adults were placed on plates containing 50 μM FUDR (RPI) and moved to fresh FUDR plates every other day until evaluation. The day before the experiment, animals were transferred to plates dried for 2 h in a laminar flow hood. On the day of the experiment, conditioned animals were exposed to butanone by streaking 5 μL of a 10% (v/v) butanone solution in ethanol on the underside of the lid of a 6‐cm plate, and incubated in a humidified chamber for 60 min at 20 °C. After conditioning, animals were washed off the plate using 1 mL of S‐basal +0.05% PEG‐8000 and transferred into 1.5‐mL tubes to settle for approximately 2 min. A 20 μL aliquot of the settled pellet was then placed at the origin of a 10‐cm chemotaxis plate containing butanone and ethanol. Chemotaxis plates were prepared by spotting 2 μL of 2% sodium azide onto the locations designated for butanone or ethanol. Plates were left for approximately 10 min to allow the sodium azide to absorb into the agar. Subsequently, 2 μL of ethanol or 10% butanone (v/v) were added to the respective spots on each plate. The same procedure was followed for non‐conditioned control animals. Excess moisture at the deposition site was gently absorbed using a folded tissue to release animals onto the plate for chemotaxis. After 1 h of chemotaxis at 20 °C, plates were transferred to a 4 °C cold room to immobilize the animals until scoring.

The learning index was calculated as the difference between the chemotaxis index (for butanone‐conditioned animals) and the naive index (for non‐conditioned animals), where each index corresponds to [(number of animals in butanone—number of animals in ethanol)/total number of animals not remaining at the origin].

The learning assay is highly sensitive to a variety of environmental conditions. Therefore, all the relevant controls are conducted as part of the same experiment and comparisons are made when the experimental conditions are matched.

### 4.8 Thrashing

Thrashing was assessed by counting the number of body bends made by each animal in 20 μL of M9 buffer over a 30‐s period. The assay was performed on at least 20 animals per condition. Each animal was placed individually in a fresh drop of M9 on a new NGM plate without bacteria, using a platinum wire and avoiding the transfer of bacteria from the plate on which the animal had been grown. Animals were allowed to acclimate for 1 min before counting, and the procedure was repeated for all animals. Although thrashing was scored manually, individual conditions were verified by video microscopy, confirming consistent thrashing behavior. Animals were age‐synchronized via standard hypochlorite treatment of gravid hermaphrodites to isolate eggs, which were allowed to hatch overnight in S‐basal supplemented with 0.05% PEG‐8000. Approximately 150 synchronized L1 larvae per condition were transferred to 6‐cm NGM plates and incubated at 20 °C until they reached the developmental stage required for each assay. For aging studies, Day 1 adults were either transferred to plates containing 50 μM FUDR (RPI) and moved to fresh FUDR plates every other day until evaluation or transferred daily to fresh plates without FUDR using a platinum wire. For details on the preparation of animals for this assay under CR conditions, please see Section 4.5 of the Materials and Methods.

### 4.9 Movement

Spontaneous movement was assessed by counting the number of body movements (sinusoidal oscillations and spontaneous reversals) over 30‐s intervals by direct observation under a stereomicroscope. Animals were prepared for evaluation as in the thrashing assay.

### 4.10 Osmotic Stress Resistance Assay

For the osmotic stress resistance assay, 30 synchronized animals at Day 1, 5, or 10 of adulthood were manually transferred using a platinum wire to NGM plates containing high salt concentration (400 mM NaCl) and seeded with *E. coli* OP50. Plates were incubated at 20 °C, and survival was assessed after 24 h by gently prodding with a platinum wire. Animals that failed to respond were scored as dead. Treatment with DMSO (vehicle) or 10 nM ADIOL was initiated at the L4 stage and discontinued at the time of transfer to the osmotic stress plates. To assess animals on Days 5 and 10 of adulthood, they were transferred daily to fresh FUDR‐free plates using a platinum wire until reaching the corresponding stages.

### 4.11 Benzaldehyde Chemotaxis

The benzaldehyde chemotaxis assay was performed as previously described (Lemieux et al. 2023). The benzaldehyde chemotaxis index was calculated as (number of animals in benzaldehyde—number of animals in ethanol)/(total number of animals). To prepare the animals for the assay, gravid hermaphrodites were age‐synchronized via standard hypochlorite treatment to isolate eggs, which were allowed to hatch overnight in S‐basal supplemented with 0.05% PEG‐8000. Approximately 150 synchronized L1 larvae per condition were transferred to 6‐cm NGM plates and incubated at 20 °C until they reached the developmental stage required for each assay. For aging studies, Day 1 adults were placed on plates containing 50 μM FUDR (RPI) and moved to fresh FUDR plates every other day until evaluation.

### 4.12 Lifespan

For lifespan assessment, animals synchronized by egg‐laying instead of bleaching were grown until Day 1 of adulthood on 6‐cm NGM plates seeded with *E. coli* OP50. From that day onward, survival was monitored daily until no live adults remained. Animals were scored as dead if they failed to respond when gently prodded with a platinum wire. In all assays, animals were transferred to plates containing 50 μM FUDR from Day 1 to 8 of adulthood, being moved to fresh plates every other day. For details on the preparation of animals for this assay under CR conditions, please see Section 4.5 of the Materials and Methods.

### 4.13 RT‐qPCR

Approximately 300 synchronized L1 larvae were transferred onto 6‐cm NGM plates per replicate and allowed to develop to the L4 stage. Worms treated with DMSO (vehicle) or F17 were exposed to the compounds overnight prior to reaching the L4 stage. Total RNA was extracted from these animals using TRIzol reagent and purified with the Direct‐zol RNA Miniprep Kit (Zymo Research), including on‐column DNase treatment. First‐strand cDNA was synthesized using the ProtoScript II First Strand cDNA Synthesis Kit (New England Biolabs) with random primers.

Quantitative PCR was performed using the SsoAdvanced Universal SYBR Green Supermix (Bio‐Rad) on a C100 Touch Thermal Cycler CFX384 Real‐Time System (Bio‐Rad), in 384‐well plates. The reactions (10 μL final volume) contained 5 μL of SYBR Green Supermix, 0.5 μL of gene‐specific primers (5 μM), 1 μL of cDNA (diluted 1:4), and 3.5 μL of nuclease‐free water.

Cycling conditions followed a two‐step protocol: initial denaturation at 95 °C for 3 min, followed by 40 cycles of 95 °C for 10 s and 60 °C for 30 s, ending with a melting curve analysis. Each sample was analyzed in technical triplicates, and appropriate negative controls were included. Threshold cycle (Ct) values were obtained using CFX Manager v3.1 software (Bio‐Rad). Gene expression was normalized to the reference gene *tba‐1*. Relative expression levels were calculated using the 2^−ΔΔCt^ method. Primer sequences are provided in Table S3.

### 4.14 Determination of Trp, Kyn, Ant, and KynA by HPLC

*C. elegans* liquid cultures of 25,000 L1 animals were cultured in 30 mL of S‐medium containing *E. coli* OP50 for 41–42 h at 22°C. *daf‐2* mutants were instead grown at 20°C to prevent dauer formation. For *ad libitum* fed cultures, animals were cultured in OP50 with an initial OD600 = 9. For cultures that were fasted 2 h prior to harvest, animals were harvested after 40 h of growth by centrifugation, washed 3 times with S‐basal, then resuspended in S‐medium without OP50 supplement for 2 h. Caloric restricted cultures were grown for 24 h with OP50 supplementation at a density of OD600 = 4.5. Animals were collected by centrifugation and resuspended in S‐medium containing OP50 at a density of OD600 = 1.6 for the remaining 16 h. To prepare animals for HPLC analysis, all cultures were purified by flotation on 30% sucrose and frozen at −80°C until analysis.

Previously frozen cultures were lysed by homogenization with a bead beater and 0.5 mm ZrO beads. The lysate was clarified by centrifugation at 10,000×*g* for 10 min. An aliquot from each sample was analyzed for protein content (Bio‐Rad DC assay), and the protein in the remaining lysate was precipitated using 5% trichloroacetic acid; the lysate was again clarified by centrifugation at 16,500×*g* for 10 min. Clarified supernatants were separated using an Agilent 1200 HPLC equipped with UV absorbance and fluorescence detectors. Injections of 0.1 mL were resolved using an isocratic mobile phase (250 mM zinc acetate, pH 6.2, 4% acetonitrile) at a flow rate of 1 mL/min through a guard column, followed by a 4.6 × 150 mm C18 reverse‐phase column (Zorbax Eclipse‐Plus 3.5 μm particle size, 100 Å pore size). KP metabolites were detected using the following channels: Trp and Kyn by absorbance at 280 and 365 nm, respectively, and Ant and KynA by fluorescence (excitation 350 nm, emission 415 nm).

### 4.15 Data Visualization and Statistics

Statistical analyses and graphical representations were performed using StataSE v12 (StataCorp LP, College Station, TX, USA) and GraphPad Prism 8.0.2 (GraphPad Software, San Diego, CA, USA). Normality was assessed using the Shapiro–Wilk test, except for feeding and thrashing data, for which the D'Agostino test was used due to the high number of repeated values. To enhance clarity, the choice between reporting the mean ± standard deviation (SD) or the median ± interquartile range (IQR), as well as the specific statistical test used, is indicated in each figure legend. The significance level (*α*) was set at 0.05, except for the log‐rank test, where it was adjusted to 0.01 to reduce the risk of type I errors (false positives).

## Author Contributions

**Kaveh Ashrafi**, **George A. Lemieux**, and **Ana Guijarro‐Hernández:** conceptualization. **Ana Guijarro‐Hernández**, **Shinja Yoo**, and **George A. Lemieux:** methodology. **Ana Guijarro‐Hernández:** formal analysis. **Ana Guijarro‐Hernández**, **Shinja Yoo**, **George A. Lemieux**, **Sena Komatsu**, **Abdullah Q. Latiff**, and **Rishika R. Patil:** investigation. **Kaveh Ashrafi:** resources. **Ana Guijarro‐Hernández:** data curation. **Shinja Yoo**, **Sena Komatsu**, and **Ana Guijarro‐Hernández:** validation. **Ana Guijarro‐Hernández** and **Kaveh Ashrafi:** writing – original draft preparation. **Kaveh Ashrafi**, **Ana Guijarro‐Hernández**, **George A. Lemieux**, **Shinja Yoo**, **Sena Komatsu:** writing – review and editing. **Ana Guijarro‐Hernández:** visualization. **Kaveh Ashrafi:** supervision and funding acquisition.

## Funding

This work was supported by NIH grants R01AG046400, RF1AG068194, and the UCSF Program in Breakthrough Biomedical Research (PBBR) to Kaveh Ashrafi.

## Conflicts of Interest

The authors declare no conflicts of interest.

## Supporting information


**Figure S1:** Background on steroidogenesis, kynurenine pathway, and regulatory connections between them through ADIOL.
**Figure S2:** ADIOL regulation of pharyngeal pumping.
**Figure S3:** Unlike pharyngeal pumping, ADIOL signaling does not affect survival rate on high salt or chemotaxis ability during aging.
**Table S1:** HPLC quantification of kynurenine pathway metabolites under different nutrient states.
**Table S2:** Strains used in this study.
**Table S3:** qPCR primers used in this study.

## Data Availability

The data supporting the findings of this study are available from the corresponding author upon reasonable request.
