# Supplementary material for: Fasting and Caloric Restriction Activate an ADIOL‐NHR‐91‐Kynurenine Pathway Signaling Axis to Promote Healthspan
Source: Aging Cell. 2026 Apr 22;25(5):e70496. doi: 10.1111/acel.70496 (PMC13103470; doi:10.1111/acel.70496)
Supplement: Supplementary file 1 — Figure S1: Background on steroidogenesis, kynurenine pathway, and regulatory connections between them through ADIOL. Figure S2: ADIOL regulation of pharyngeal pumping. Figure S3: Unlike pharyngeal pumping, ADIOL signaling does not affect survival rate on high salt or chemotaxis ability during aging. Table S1: HPLC quantification of kynurenine pathway metabolites under different nutrient states. Table S2: Strains used in this study. Table S3: qPCR primers used in this study. [file ACEL-25-e70496-s001.pdf]

Fasting and Caloric Restriction Activate an ADIOL-NHR-91-Kynurenine Pathway Signaling Axis to Promote Healthspan

Ana Guijarro-Hernández[1], Shinja Yoo[1], George A. Lemieux[1], Sena Komatsu[1], Abdullah Q. Latiff[1], Rishika R. Patil[1], and Kaveh Ashrafi[1*]

1. Department of Physiology, University of California, San Francisco, San Francisco, California, USA

*Correspondence: kaveh.ashrafi@ucsf.edu (K.A.)

SUPPORTING INFORMATION

Figures S1-S3

Tables S1-S3

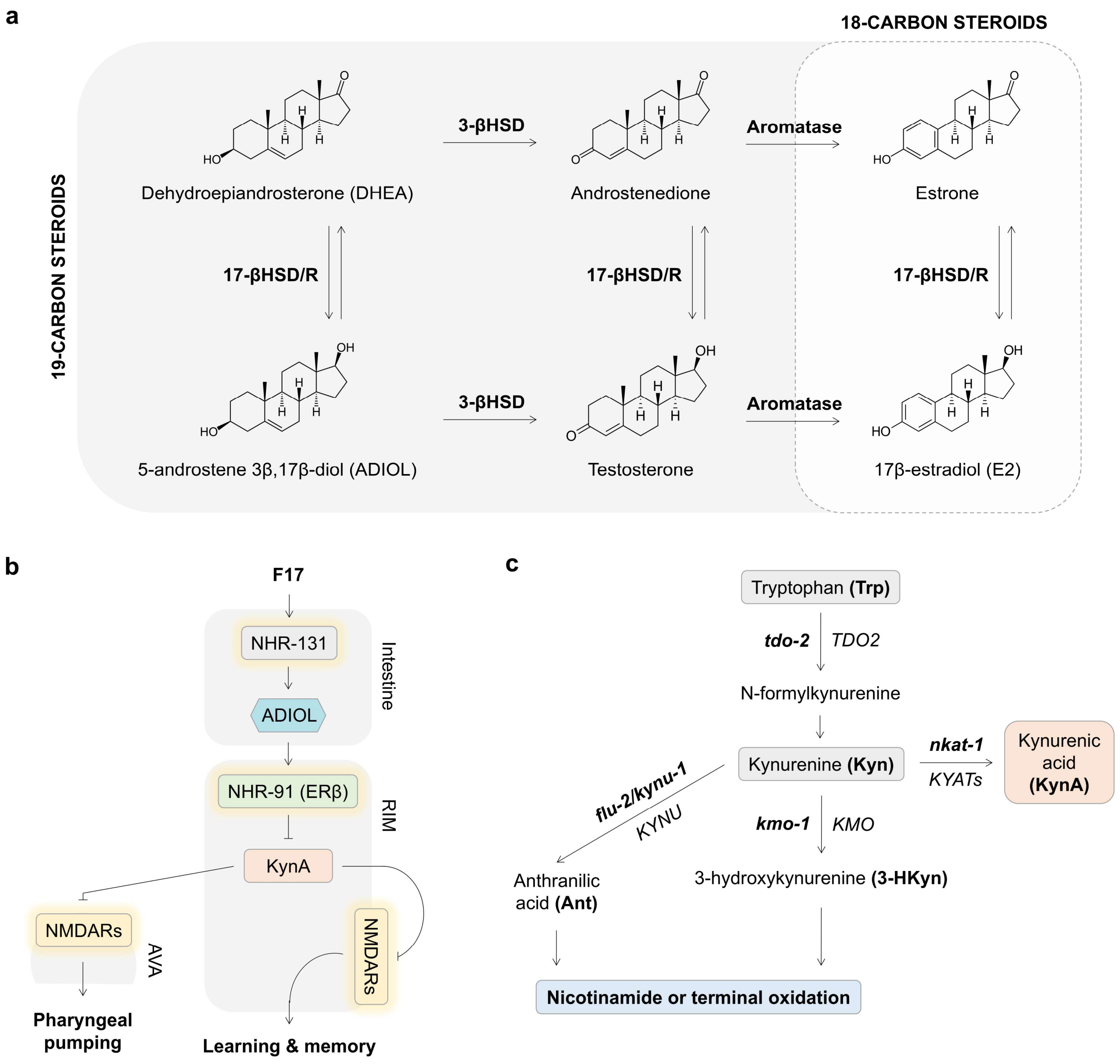

Figure S1. Background on steroidogenesis, kynurenine pathway, and regulatory connections between them through ADIOL. (**A**) 5-androstene-3β,17β-diol (ADIOL) is a 19-carbon steroid produced from dehydroepiandrosterone (DHEA), which acts as an intermediate in the synthesis of other 19-carbon steroids such as testosterone, as well as 18-carbon steroids like 17β-estradiol (E2). (**B**) F17 promotes learning and memory and pharyngeal pumping through reductions in kynurenic acid (KynA). The synthetic compound F17 activates the transcription factor NHR-131 in the intestine, leading to an increase in ADIOL production. NHR-91, a *C. elegans* ERβ-like nuclear hormone receptor is required in the RIM neuron for the learning and pharyngeal pumping effects of ADIOL. ADIOL-induced decrease in KynA activates RIM and AVA neurons dependent on NMDARs. The learning effects of KynA depend on NMDARs in the RIM neurons while KynA's effects on pumping depend on NMDARs in the AVA neurons. (**C**) Schematic of the kynurenine pathway. Tryptophan (Trp) is converted to kynurenine (Kyn) by the enzyme TDO2 (TDO-2). Kyn is the branchpoint leading to generation of KynA, 3-hydroxykynurenine (3-HKyn), or anthranilic acid (Ant) depending on the enzymatic activities of NKAT-1, KMO-1, and KYNU-1, respectively. Through several additional enzymatic steps, Ant and 3-HKyn lead to generation of substrate for *de novo* NAD+ synthesis.

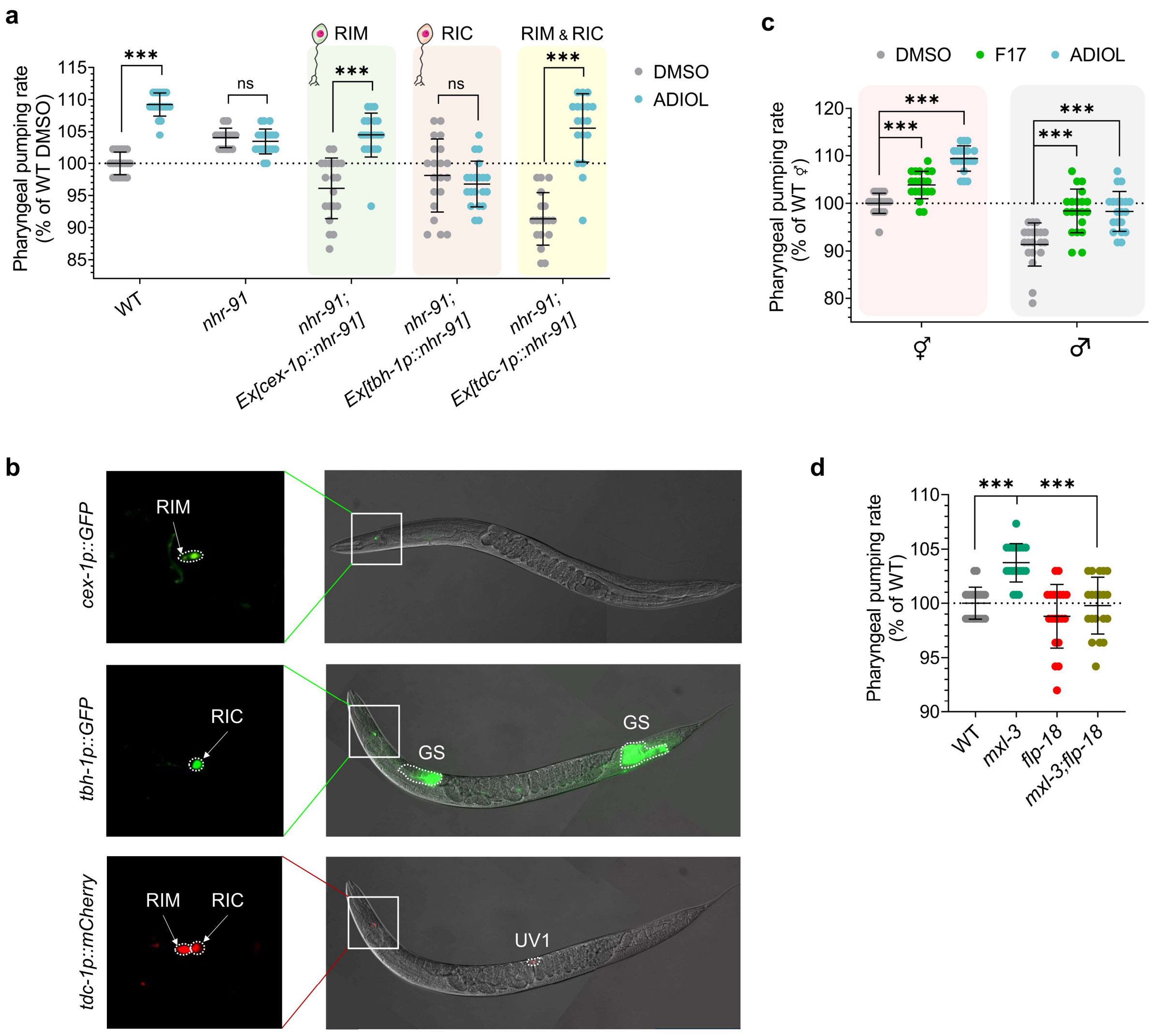

Figure S2. ADIOL regulation of pharyngeal pumping. (**A-C**) Pharyngeal pumping rate in Day 1 adults, normalized to WT controls (n = 20 animals/condition; mean ± SD). (**A**) WT, *nhr-91*, and strains in which WT *nhr-91* was reconstituted in select cells (*nhr-91*; *Ex[cex-1p::nhr-91,unc-122::GFP]*, *nhr-91; Ex[tbh-1p::nhr-91cDNA::sl2::GFP],* and *nhr-91; Ex[tdc-1p::nhr-91::sl2::GFP]*) treated with DMSO or 10 nM ADIOL from the L4 stage. Statistics: t-test (***p < 0.001, ns: non-significant). (**B**) Expression patterns of *cex-1*, *tbh-1*, and *tdc-1* promoter–driven transgenes in *C. elegans*. Images show previously reported expression patterns: *cex-1p* drives expression in RIM neurons, *tbh-1p* in RIC neurons and gonadal sheath cells (GS), and *tdc-1p* in both RIM and RIC neurons as well as uterine UV1 cells. None of these lines exhibit aberrant expression patterns. (**C**) WT hermaphrodites and males treated with DMSO, 2.5 µM F17 or 10 nM ADIOL from the L4 stage. Statistics: one-way ANOVA followed by Dunnett's test (***p < 0.001). (**D**) WT, *mxl-3*, *flp-18*, and *mxl-3*; *flp-18* strains. Statistics: one-way ANOVA followed by Tukey's multiple comparisons test (***p < 0.001).

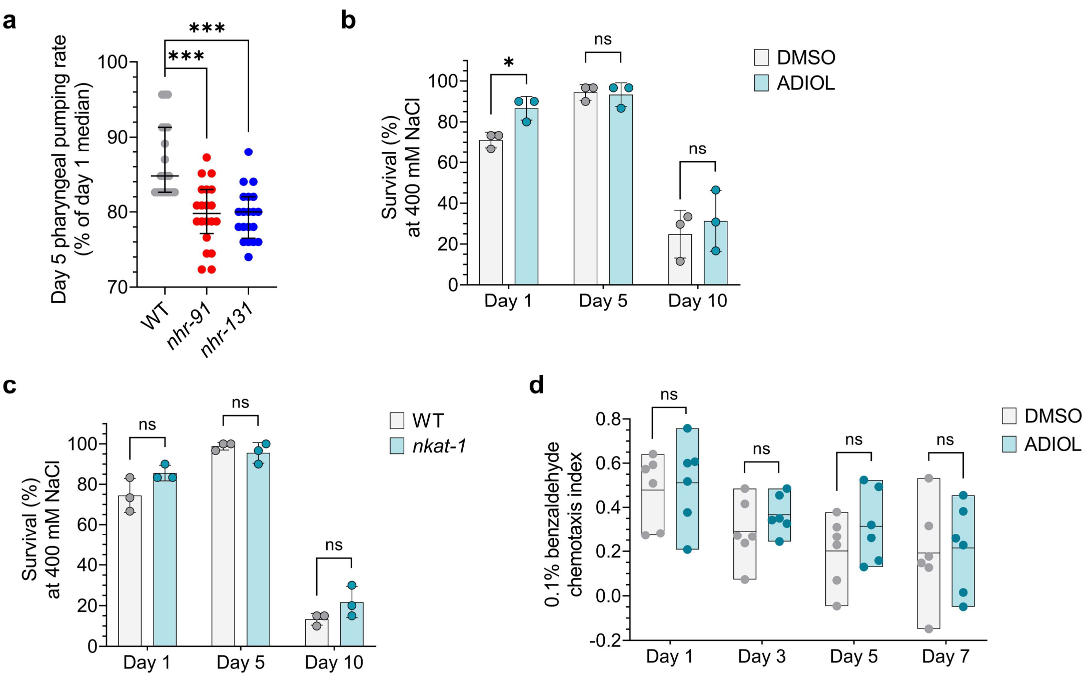

Figure S3. Unlike pharyngeal pumping, ADIOL signaling does not affect survival rate on high salt or chemotaxis ability during aging. (**A**) Pharyngeal pumping rate in WT, *nhr-91*, and *nhr-131* animals on Days 1 and 5 of adulthood (n = 20 animals/condition). Median ± IQR values shown relative to Day 1 WT adults. Animals were treated with 50 µM FUDR starting on Day 1. Statistics: Kruskal-Wallis test followed by Dunn's test with Bonferroni correction for multiple comparisons (*p < 0.001). (**B, C**) Survival after 24-h exposure to 400 mM NaCl, normalized to WT controls on Day 1. Mean ± SD shown (n = 3; ~30 animals/replicate). No FUDR was used in this assay. Statistics: t-test (***p < 0.001, **p < 0.01, ns: non-significant). (**B**) WT animals treated with DMSO or 10 nM ADIOL from the L4 stage. (**C**) WT and *nkat-1* animals. (**D**) Chemotaxis index toward 0.1% benzaldehyde in WT animals treated with DMSO or 10 nM ADIOL on Days 1, 3, 5, and 7 of adulthood. Treatment began at the L4 stage, with 50 µM FUDR from Day 1. Mean, minimum, and maximum shown (n = 3; ~38-95 animals/replicate). Statistics: t-test (ns: non-significant).

Table S1. HPLC quantification of kynurenine pathway metabolites under different nutrient states.

		WT			*nhr-91(tm4713)*			*daf-2(e1370)*
		**Well-fed**	**2h-fast**	**CR**	**Well-fed**	**2h-fast**	**CR**	**Well-fed**
**TRP (µmol/mg)**	**1**	31.81	20.98	15.27	31.46	28.74	25.26	14.87
	**2**	27.88	21.72	15.54	33.57	23.92	20.10	12.75
	**3**	26.43	22.58	19.89	27.82	26.14	22.23	14.14
	**4**	28.61	26.50	21.28	30.21	31.13	25.82	15.43
	**5**	35.61	23.78	15.25	38.98	32.16	25.03	-
	Mean	*30.07*	*23.11*	*17.45*	*32.41*	*28.42*	*23.69*	*14.30*
**KYN (nmol/mg)**	**1**	654.72	786.95	309.95	543.45	565.87	218.14	118.91
	**2**	568.88	774.49	361.32	543.65	575.83	267.53	158.02
	**3**	614.68	755.33	350.53	571.79	560.90	274.42	162.61
	**4**	650.78	717.14	204.39	528.17	610.97	275.94	134.19
	**5**	735.97	765.39	198.85	481.29	612.01	255.88	-
	Mean	*645.01*	*759.86*	*285.01*	*533.67*	*585.12*	*258.38*	*143.43*
**KYNA (pmol/mg)**	**1**	0.73	0.18	0.29	0.41	0.33	0.37	0.14
	**2**	0.51	0.18	0.21	0.76	0.28	0.22	0.13
	**3**	0.52	0.27	0.28	0.55	0.41	0.26	0.15
	**4**	0.39	0.34	0.33	0.70	0.45	0.42	0.15
	**5**	0.59	0.22	0.28	0.43	0.25	0.41	-
	Mean	*0.55*	*0.24*	*0.28*	*0.57*	*0.34*	*0.33*	*0.14*
**ANT (pmol/mg)**	**1**	1094.74	1577.03	1191.64	625.01	935.84	728.14	957.83
	**2**	1015.08	1539.06	1077.70	772.62	867.55	690.07	834.37
	**3**	991.95	1491.31	1026.94	544.65	847.22	694.17	761.87
	**4**	1074.02	1610.10	1063.45	586.34	985.80	819.92	875.52
	**5**	1186.03	1439.86	855.79	567.81	907.07	698.65	-
	Mean	*1072.36*	*1531.47*	*1043.11*	*619.29*	*908.70*	*726.19*	*857.40*

Table S2. Strains used in this study.

Strain name (in text)	Genotype
Wild-type/WT	WT
nhr-91	*nhr-91*(tm4713) X
nhr-131	*nhr-131*(tm1376) V
nhr-91;Ex[cex-1p::nhr-91]	*nhr-91*(tm4713) X; *Ex[cex-1p::nhr-91, unc-122::GFP]*
nhr-91;Ex[tbh-1p::nhr-91]	*nhr-91*(tm4713) X; *Ex[tbh-1p::nhr-91cDNA::SL2::GFP]*
nhr-91;Ex[tdc-1p::nhr-91]	*nhr-91*(tm4713) X; *Ex[tdc-1p::nhr-91cDNA::SL2::GFP]*
daf-2	*daf-2*(e1370) III
daf-2; nhr-91	*daf-2*(e1370) III; *nhr-91*(tm4713) X
daf-2; nhr-131	*daf-2*(e1370) III; *nhr-131*(tm1376) V
mxl-3	*mxl-3*(ok1947) X
mxl-3; nhr-91	*mxl-3*(ok1947) X; *nhr-91*(tm4713) X
mxl-3; nhr-131	*mxl-3*(ok1947) X; *nhr-131*(tm1376) V
flp-18	*flp-18*(gk3063) X
mxl-3; flp-18	*mxl-3*(ok1947) X; *flp-18*(gk3063) X
rict-1(mg360)	*rict-1*(mg360) II
rict-1(mg360); nhr-91	*rict-1*(mg360) II; *nhr-91*(tm4713) X
rict-1(ft7)	*rict-1*(ft7)
rict-1(ft7); nhr-91	*rict-1*(ft7); *nhr-91*(tm4713) X
cyp-44A1	*cyp-44A1*(ok216) II
cyp-13A4	*cyp-13A4*(tm7443) II
F12E12.11	*F12E12.11*(ft1005) II
nkat-1	*nkat-1*(ok566) X
kmo-1	*kmo-1*(tm4529) V
eat-2	*eat-2*(ad465) II
eat-2; nhr-91	*eat-2*(ad465) II; *nhr-91*(tm4713) X

Table S3. qPCR primers used in this study.

Gene	F/R	Sequence	Tm	Genomic length	Mature length
cyp-44A1	F	CTCGAATCTGCTGGTCAAATA	60	200	156
	R	TGTTGAGAACAGACGGAATATC	60		
cyp-13A4	F	GCTCTACGAATGTACCCTTTAG	60	214	120
	R	AAGTGTCCATGTATCCACTTG	60		
cyp-33C2	F	CCCGTTCTCAGTTGGAAA	59	197	146
	R	GGGCTCCATTGCTCTTATC	60		
cyp-33C5	F	GAATGAGACACTTGGTGGAG	60	256	207
	R	GGCTTTCCTCGGAATCAAA	60		
cytb-5.1	F	CTGACGCTAGGCATATGAAG	60	166	112
	R	TTATCCTGTTCGGTGGTAGA	60		
F12E12.11	F	AGTCTACACAGGATTCGGAG	60.4	400	127
	R	GGCAATTTCTATGGGCTGAG	60.8		
F25D1.5	F	GCTGTGATCGAAATGACTCAG	60.9	203	156
	R	GCATCTGGTGTATTGGTCAAG	61		
R05D8.9	F	CGGGTTTGGAGAAGCTATG	60	184	141
	R	TCAGCCAAGAACGCAATAA	60		
tba-1*	F	ACACTCCACTGATCTCTGC	60.9	174	129
	R	CAGCCATGTACTTTCCGTG	60.5		

*Reference gene
